# Optimizations for Computing Relatedness in Biomedical Heterogeneous Information Networks: SemNet 2.0

**DOI:** 10.3390/bdcc6010027

**Published:** 2022-03-01

**Authors:** Anna Kirkpatrick, Chidozie Onyeze, David Kartchner, Stephen Allegri, Davi Nakajima An, Kevin McCoy, Evie Davalbhakta, Cassie S. Mitchell

**Affiliations:** 1 Laboratory for Pathology Dynamics, Georgia Institute of Technology and Emory University, Atlanta, GA 30332, USA; 2 School of Mathematics, Georgia Institute of Technology, Atlanta, GA 30332, USA; 3 School of Computer Science, Georgia Institute of Technology, Atlanta, GA 30332, USA; 4 Department of Biomedical Engineering, Georgia Institute of Technology and Emory University, Atlanta, GA 30332, USA; 5 Machine Learning Center at Georgia Tech, Georgia Institute of Technology, Atlanta, GA 30332, USA

**Keywords:** HeteSim, ULARA, SemNet, Alzheimer’s disease, natural language processing, machine learning, text mining, biomedical knowledge graph, relatedness, rank aggregation

## Abstract

Literature-based discovery (LBD) summarizes information and generates insight from large text corpuses. The SemNet framework utilizes a large heterogeneous information network or “knowledge graph” of nodes and edges to compute relatedness and rank concepts pertinent to a user-specified target. SemNet provides a way to perform multi-factorial and multi-scalar analysis of complex disease etiology and therapeutic identification using the 33+ million articles in PubMed. The present work improves the efficacy and efficiency of LBD for end users by augmenting SemNet to create SemNet 2.0. A custom Python data structure replaced reliance on Neo4j to improve knowledge graph query times by several orders of magnitude. Additionally, two randomized algorithms were built to optimize the HeteSim metric calculation for computing metapath similarity. The unsupervised learning algorithm for rank aggregation (ULARA), which ranks concepts with respect to the user-specified target, was reconstructed using derived mathematical proofs of correctness and probabilistic performance guarantees for optimization. The upgraded ULARA is generalizable to other rank aggregation problems outside of SemNet. In summary, SemNet 2.0 is a comprehensive open-source software for significantly faster, more effective, and user-friendly means of automated biomedical LBD. An example case is performed to rank relationships between Alzheimer’s disease and metabolic co-morbidities.

## 1. Introduction

Biomedical research, like the human body itself, is a complex network of interrelated concepts and relationships that make up a greater whole. There are more than 33 million abstracts and counting in PubMed, one of the largest and most widely used databases and search engines for biomedical research [1]. Many researchers use PubMed, or similar databases, to look up information using specific keywords. However, it is impossible to manually read and synthesize all articles across all related topics. The goal of literature-based discovery (LBD), founded by Dr. Swanson in 1986 [2], is concentrating and concatenating conclusions between disparate sources of information to both improve existing insights as well as generate new insights. The field of LBD attempts to capture knowledge from biomedical text and integrate it in a way that makes discovery of new knowledge possible. In Henry et al. [3], LBD techniques were used to discover lecithin-cholesterol acyltransferase (LCAT) as a proposed therapeutic target for cardiac arrest, a target that was later supported via in vivo studies. Additionally, LBD was used to identify repurposed drugs for the COVID-19 pandemic [4]. While LBD has the potential to be truly transformative, challenges remain to optimize the underlying text mining methodology as well as to make LBD more accessible to domain specialists and clinicians. The presented work optimizes LBD by improving the efficiency and efficacy of LBD in an interactive, open-source Python-based framework called SemNet 2.0.

### 1.1. Automating the LBD Process

The first step in the LBD process is to model the connections between biomedical concepts in a medium where both humans and computers can easily work with the data. Heterogeneous information networks, or more specifically biomedical concept graphs, provide an exceptional scaffold and starting point for LBD. Modeling biomedical relationships using a graph structure is not ubiquitous in the LBD field, though it is common and wrought with research potential. Various methods, including those described in Cameron et al. [5], Crichton el al. [6], and Sang et al. [7], have used graph-based approaches to perform LBD to great success (all of which vary in how the graphs are constructed and analyzed). Simply put, a biomedical concept graph is the most intuitive and flexible representation available to model semantic predications, especially given the heterogeneous nature of the data and overall direction the LBD field is currently moving. As a brief aside, the terminology used to describe these graphs is borrowed from graph theory and social network analysis; biomedical concepts are referred to as “nodes” and connections between concepts are referred to as “edges”. In the context of the present study, this data representation is built as a directed graph in which each node corresponds to a Unified Medical Language System (UMLS) biomedical concept (Alzheimer’s disease, insulin, COVID-19, etc.) and each directed edge encodes a UMLS predication (inhibits, treats, causes, etc.) between a source and target. Additionally, each node has an associated UMLS semantic type (disease or syndrome, gene or genome, therapeutic or preventive procedure, etc.). Each concept–predication–concept relationship within the graph has been extracted from biomedical article abstracts within PubMed via SemRep and stored within the MySQL-based Semantic Medline Database (SemMedDB) [8]. SemMedDB is essentially a table of subject–predicate–object triples, which are manipulated to form an approximately 300,000 node and 20,000,000 edge knowledge graph that combines standardized biomedical concepts and relationships.

The second major step in LBD is using natural language processing and machine learning techniques to identify concepts of interest that are related to a user-specified query. A user, such as a domain specialist, specifies one or more target nodes of interest, which defines the topic upon which the user wishes to discover and ultimately rank related concepts from the literature. The target is analogous to a user entering a keyword to lookup an article in PubMed. Then, using a few other user inputs to constrain the queried biomedical knowledge graph results, such as node type (pharmacologic substance; gene or genome; disease or syndrome, etc.) and/or relation type (treats, affects, inhibits, etc.), the algorithm finds all the related source nodes by examining metapaths in the graph. A metapath is a series of sequential node and relationship types that tie the user-specified target node to the related source nodes identified in the graph.

The third major step in LBD is using machine learning and mathematical optimization to rank the importance of the identified source nodes to the target node. There are various methods of ranking concepts in heterogeneous information networks, often stemming from an assortment of domains [9–11]. The most common methods utilize the graph’s metapaths to perform statistical analysis on one more more features of interest. Example features may be simple node or path counts, or they may be more complex, such as the HeteSim metric, which examines metapath similarities [12]. Finally, a ranking algorithm uses the feature scores to provide a final ranking that can provide important context to a domain scientist. That is, of all the identified related concepts of interest, which ones are most important to the user-specified target?

### 1.2. Overview of SemNet

SemNet was the first Python-based open-source software that enabled all three steps of LBD to be performed using the 33 plus million biomedical abstracts in PubMed [13]. SemNet will be briefly introduced here and is fully described in the original published work by Sedler and Mitchell [13]. First, SemNet had the initial task of building the knowledge graph derived from SemMedDB’s semantic relationships and storing it, which it did via an interactive graph database management system called Neo4j. Second, SemNet used py2neo to query the biomedical concept graph constructed in Neo4j to compute metapaths. Third, SemNet adopted a version of the unsupervised learning algorithm for rank aggregation, ULARA, published by Klementiev and colleagues [14] to perform the calculations for feature rankings, including the computation of the HeteSim score. In summary, SemNet enabled a user to easily input a few targets and subsequently retrieve source node importance rankings using machine learning to process millions of biomedical concepts.

### 1.3. Improving LBD Efficiency and Efficacy with SemNet 2.0

SemNet laid an important foundation for making LBD accessible and usable for domain researchers. However, SemNet simulations were extremely slow, even when performed on high-end servers. The slowness and amount of required computation also limited the amount of detail that could be studied in SemNet. Namely, it limited the length of metapaths that could be ranked (e.g., maximum possible path length for ranking calculation was equal to two); this limit was problematic for a domain researcher wishing to examine more nuanced related concepts that would likely have a longer path length. The present study was largely motivated by the need to enhance SemNet to improve its computation speed, usability and utility. From this point forward, the original SemNet will be referred to as SemNet version 1. The present study performs a full evaluation of speed bottlenecks in SemNet version 1 and proposes and evaluates alternative solutions. The research process to improve speed led to additional mathematical scrutiny of the utilized HeteSim metric and ULARA algorithms in SemNet version 1. Thus, the present study includes both a presentation of optimized mathematical solutions as well as changes to algorithmic and data handling frameworks to increase overall speed. Three major technical improvements were made to create SemNet version 2 (also known as SemNet 2.0): (1) a randomized approximation algorithm for estimating HeteSim scores to improve HeteSim calculation speed; (2) a re-engineered knowledge graph framework that removed reliance on Neo4j to improve metapath and feature computation speed; (3) an improved implementation of the adopted ULARA ranking algorithm.

The first major improvement focused on the efficiency of algorithms utilizing the HeteSim metric. HeteSim-based similarity scoring on heterogeneous information networks has been successfully applied to multiple biomedical research problems [15–20]; therefore, the implementation of a faster HeteSim scoring algorithm will have the potential for significant benefit to the biomedical research community. The main investigative line for algorithm improvements involves approximation algorithms using randomness. An approximation algorithm is a unique algorithm which returns a value within a specified error (generally additive or multiplicative) of the true answer, with some known or bounded probability. The power of approximation algorithms lies in their ability, for some problems, to provide a fast approximation to a solution even when computing the exact solution requires exponential time (assuming (*P* ≠ *NP*)). Though approximation algorithms have existed in the literature for some time, Garey, Graham, and Ullman [21] and Johnson [22] both introduced the idea formally in 1973 and 1974, respectively. Since then, the computer science and combinatorics literature has featured many advancements in the field of randomized approximation algorithms. For an overview of basic techniques and more recent results, see [23–25].

The second major improvement focused on re-engineering the graph data structure to remove query processing bottlenecks and improve overall performance via faster data accessibility. SemNet version 1 uses Neo4j, an efficient graph database management system that employs a specialized declarative query language (Cypher) optimized for graphs, to store and query the biomedical concept graph [26]. At first glance, the choice to use Neo4j is intuitive. It is custom designed to deal with graphs akin to the one SemNet builds, and it has been used before in similar projects to great success [9]. Nonetheless, constantly querying an externally accessible database to run the HeteSim algorithm, even with the use of multi-threading, proved much slower than desired. This outcome prompted an investigation into alternatives to Neo4j. The evaluated alternative is a locally stored Python nested dictionary graph representation, a data structure that lacks the appealing interfaces of Neo4j but has greatly improved data handling speeds.

The third major improvement focused on the ULARA ranking algorithm. Careful mathematical investigation of ULARA led to the identification of a pertinent flaw in the originally published ULARA algorithm [14]. As noted above, SemNet version 1 had adopted ULARA for aggregating HeteSim scores over multiple metapaths. Fortunately, the specific implementation of ULARA to SemNet version 1 minimized the impact of the identified ULARA flaw on SemNet version 1 results. Nonetheless, a full and precise solution was necessary to correctly fix ULARA and improve the produced rankings. Section 2.1 explains the flaw in the original ULARA [14] and proposes an alternative, which was implemented in SemNet version 2.

The mathematics of the SemNet version 2 improvements are derived in full in subsequent sections. Beyond the mathematics, real-world examples and user studies are used to showcase the improvements and power of SemNet version 2.

### 1.4. Use Case Example: Alzheimer’s Disease and Metabolism

SemNet version 2 was primarily developed for interactive, multi-factorial and multi-scalar relationship exploration in biomedical science and health care. For this study, the primary target node, Alzheimer’s disease (AD), was chosen to compare performance of the original SemNet (i.e., SemNet version 1) to the developed SemNet version 2. AD was chosen due to its large degree of connectivity, multi-factorial and heterogeneous nature, and growing relevance in health care—a byproduct of increasing AD deaths and an aging global population [27,28]. AD is traditionally characterized by its tau and amyloid beta protein deposition in neurofibrillary tangles, brain atrophy, and eventual cognitive decline [3]. As researchers delved deeper into the disease, the breadth of risk factors across various domains, such as pharmaceuticals, antecedent disease, psychological profile, and lifestyle, has further increased overall complexity of AD investigation [27,29,30]. This complexity is exacerbated by the difficulty of defining AD sub-populations, a problem that impacts clinical trial patient selection and therapeutic evaluation [31]. Given AD’s heterogeneous nature, traditional bioinformatics solutions struggle where the SemNet framework thrives. SemNet version 2 is optimized to work with heterogeneous data, drawing from literature across all biomedical domains to provide concept rankings. The flexibility, efficacy, and efficiency of SemNet version 2 is evaluated using AD as a case study. Thus, Alzheimer’s disease (CUI: C0002395) is chosen as the primary target to three diverse sources: insulin (CUI: C0021641), hypothyroidism (CUI: C0020676), and amyloid (CUI: C0002716). Amyloid was chosen as a known “control”, where the relationship between amyloid and Alzheimer’s disease is well known and validated; therefore, amyloid has many paths and metapaths connecting it to AD [32]. Insulin and hypothyroidism were chosen to assess a newer hypothesis that metabolic syndromes may play a significant role in the onset risk or outcome of AD [31,33]. The nodes of insulin and hypothyroidism have sufficient connections to AD to be considered relevant but are distant enough, domain wise, to showcase SemNet’s flexibility in exploring more nuanced, and lesser cited multi-factorial disease etiology [34,35].

### 1.5. Definitions and Mathematical Preliminaries

In this section, we will formally define a schema and a knowledge graph/heterogeneous information network. A schema tells us which node and edge types may be present in our knowledge graph, while the knowledge graph tells us which relations apply to specific concepts nodes.

**Definition 1**. *A schema S=(A,R) is a set A of node types and a set R of relations*. *Each relation R∈R has a source type A∈A and a target type B∈A*.

**Definition 2**. *Let S=(A,R) be a schema with |A|>1*. *Then, a heterogeneous information network (also called a knowledge graph) is a directed graph G* = (*V*, *E*) *with an object type mapping function φ:V→A and a link type mapping function ψ:E→R*. *If e* = (*u*, *v*) ∈ *E, then the source type of ψ*(*e*) *must be φ*(*u*) *and similarly the target type of ψ*(*e*) *must be φ*(*v*).

Relations are a key concept in understanding knowledge graphs. We may understand both individual edges and entire metapaths as relations. We start by defining the simplest relation, the self relation.

**Definition 3**. *The relation I is the self-relation*. *So, $a\overset{I}{\to }b$ if and only if a* = *b*. *We also define the function δ by δ*(*a*, *b*) = 1 *if a* = *b and δ*(*a*, *b*) = 0 *otherwise*.

We now define our primary object of study: the metapath. Note that the metapath may be viewed as a list of node and edge types or as the relation equivalent to the composition of all individual relations in the metapath.

**Definition 4**. *Let* S=(A,R) *be a schema*. *Then, a metapath* P *is a sequence of node and edge types, denoted* $A_{1}\overset{R_{1}}{\to }A_{2}\overset{R_{2}}{\to }\ldots \overset{R_{l}}{\to }A_{l+1}$, *with* $A_{i}\in A$ *and* $R_{i}\in R$. *The length of P is l*. *Note that a metapath may also be understood as the composition of the relations given by its metaedges: R* = *R*_1_ ◦ *R*_2_ ◦⋯◦ *R*_*l*_. *Let p* = *a*_1_*a*_2_ … *a*_*l*+1_ *with a*_*i*_ ∈ *V and* (*a*_*i*_, *a*_*i*+1_) ∈ *E be a path in G*. *Then, p is a path instance of the metapath P if φ*(*a*_*i*_) = *A*_*i*_∀*i* ≤ *l* + 1 *and ψ*((*a*_*i*_, *a*_*i*+1_)) = *R*_*i*_∀*i* ≤ *l*. *We denote the fact that p is a path instance of P by p∈P*.

Given these definitions, we are nearly ready to define the function of interest: *HeteSim*, which was defined by Shi et al. [12]. We start by defining a function *h* which is a non-normalized version of HeteSim.

**Definition 5**. *Let l* > 0. *Let $P=A_{1}\overset{R_{1}}{\to }A_{2}\overset{R_{2}}{\to }\ldots \overset{R_{l}}{\to }A_{l+1}$*. *Let φ*(*s*) = *A*_1_ *and φ*(*t*) = *R*_*l*+1_. *Then the non-normalized HeteSim score between s and t with respect to the relevance path P is defined recursively as follows*. *When R*_1_ ◦ *R*_2_ ◦ … *R*_*l*_ ≠ *I*,

$$h\left(s,t|R_{1}\circ R_{2}\circ \cdots \circ R_{l}\right)=\frac{1}{\left|O\left(s|R_{1}\right)\right|\left|I\left(t|R_{l}\right)\right|}\underset{a\in O\left(s|R_{1}\right)}{\sum }\underset{b\in I\left(t|R_{l}\right)}{\sum }h\left(a,b|R_{2}\circ R_{3}\circ \cdots \circ R_{l-1}\right),$$

*where O*(*s*|*R*_1_) *is the set of out-neighbors of node s based on relation R*_1_*, and I*(*t*|*R*_*l*_) *is the set of in-neighbors of node t based on the relation R*_*l*_. *In the base case, we define*

h(a,b∣I)=δ(a,b).

*Note that this definition only works for relevance paths of even length*. *We will need an extension for paths of odd length*.

*We briefly explain the definition of HeteSim for odd paths here*. *For more detail, see Shi et al*. [12].

The basic idea to define *h* for paths of odd length is to transform those paths into paths of even length. Suppose we have a relevance path of odd length $P=A_{1}\overset{R_{1}}{\to }A_{2}\overset{R_{2}}{\to }\ldots \overset{R_{l}}{\to }A_{l+1}$. We now modify P by adding a new object type *E* and two new relation types *R*_*E*_ and *R*_*F*_. We then define $P^{\prime }=A_{1}\overset{R_{1}}{\to }A_{2}\overset{R_{2}}{\to }\ldots \overset{R_{\frac{l+1}{2}-1}}{\to }A_{\frac{l+1}{2}}\overset{R_{E}}{\to }E\overset{R_{F}}{\to }A_{\frac{l+1}{2}+1}\overset{R_{\frac{l+1}{2}+1}}{\to }\ldots \overset{R_{l}}{\to }A_{l+1}$. Additionally, in the underlying graph *G*, for any edge *g* = (*u*, *v*) with $\psi (g)=R_{\frac{l+1}{2}}$, we add a new node, *E*_*g*_ and 2 new edges: *e*_1_ = (*u*, *E*_*g*_) and *e*_2_ = (*E*_*g*_, *v*). We additionally assign *φ*(*E*_*g*_) = *E*, *ψ*(*e*_1_) = *R*_*E*_, and *ψ*(*e*_2_) = *R*_*F*_. This procedure allows us to transform any odd path into an even path, giving a definition for the non-normalized HeteSim score *h* for odd length paths.

As a final step, HeteSim is normalized so that the normalized score for any two nodes lies in the interval [0, 1]. To do so, we will cast the problem in the language of transition matrices.

**Definition 6**. *Given a relation $A\overset{R}{\to }B$, let W*_*AB*_ *be an adjacency matrix between type A and type B*. *Let U*_*AB*_ *be W*_*AB*_ *normalized along each row vector*. *That is, U*_*AB*_ *is the transition probability matrix A* → *B based on relation R where each allowed transition is given equal probability*. *Similarly, let V*_*AB*_ *be a normalized form of the matrix W*_*AB*_*, this time normalized along its column vectors*. *So, V*_*AB*_ *is the transition probability matrix for B* → *A based on relation R*^−1^. *Note that $U_{AB}=V_{BA}^{T}$*.

**Definition 7**. *Given a metapath $P=A_{1}\overset{R_{1}}{\to }A_{2}\overset{R_{2}}{\to }\ldots \overset{R_{l}}{\to }A_{l+1}$, the reachable probability matrix PM for that metapath is given by*

$$PM_{P}=U_{A_{1}A_{2}}U_{A_{2}A_{3}}\ldots U_{A_{l}A_{l+1}}.$$

*Note that $PM_{P}(i,j)$ gives us the probability of object i* ∈ *A*_1_ *reaching object j* ∈ *A*_*l*+1_ *under the path P, under the assumption that at each step all valid transitions have equal probability*.

The following lemma is implicit in [12], but it is stated here for clarity.

**Lemma 1**. *Let s* ∈ *A*_1_*, t* ∈ *A*_*l*+1_. *Let $P=\left(A_{1}A_{2}\ldots A_{l+1}\right)$ be a metapath*. *Then*,

$$h(s,t|P)=PM_{P_{L}}(s,:){\left(PM_{P_{R}^{-1}}(t,:)\right)}^{T},$$

*where $PM_{P_{L}}(a,:)$ is used to denote the ath row of the matrix $PM_{P}$, and $P=P_{L}P_{R}$ is the decomposition of P into two paths of equal length*.

**Proof**. First, we only need to prove this result for even values of *l*. We proceed by induction. In the base case, we have *l* = 0. This is the trivial metapath, and its corresponding relation is the self relation. We have

h(s,s)=δ(s,s)=1,

and

$$PM_{P_{L}}(s,:){\left(PM_{P_{R}^{-1}}(s,:)\right)}^{T}=1\times 1=1.$$

Therefore, the base case holds.

For the induction step, let *k* ≥ 2 be an even integer. Assume that the lemma holds for all metapaths of length *k*. We will prove the lemma for paths of length *k* + 2. Beginning with the definition of *h*, we have

$$h\left(s,t|R_{1}\circ R_{2}\circ \cdots \circ R_{k+2}\right)\;=\frac{1}{\left|O\left(s|R_{1}\right)\right|\left|I\left(t|R_{k+2}\right)\right|}\underset{a\in O\left(s|R_{1}\right)}{\sum }\underset{b\in I\left(t|R_{k+2}\right)}{\sum }h\left(a,b|R_{2}\circ \cdots \circ R_{k+1}\right)\;=\frac{1}{\left|O\left(s|R_{1}\right)\right|\left|I\left(t|R_{k+2}\right)\right|}\underset{a\in O\left(s|R_{1}\right)}{\sum }\underset{b\in I\left(t|R_{k+2}\right)}{\sum }PM_{P_{L}^{\prime }}(a,:){\left(PM_{{\left(P^{\prime }\right)}_{R}^{-1}}(b,:)\right)}^{T},$$

where $P^{\prime }=R_{2}\circ \cdots \circ R_{k+1}$, and the second equality follows from the induction hypothesis. Recalling the interpretation of $PM_{P}$ as the product of transition matrices, we see

$$\frac{1}{\left|O\left(s|R_{1}\right)\right|\left|I\left(t|R_{k+2}\right)\right|}\sum_{a\in O\left(s|R_{1}\right)}\sum_{b\in I\left(t|R_{k+2}\right)}PM_{P_{L}^{\prime }}(a,:){\left(PM_{{\left(P^{\prime }\right)}_{R}^{-1}}(b,:)\right)}^{T}\;=\sum_{a\in O\left(s|R_{1}\right)}\frac{1}{\left|O\left(s|R_{1}\right)\right|}PM_{P_{L}^{\prime }}(a,:)\sum_{b\in I\left(t|R_{k+2}\right)}\frac{1}{\left|I\left(t|R_{k+2}\right)\right|}{\left(PM_{{\left(P^{\prime }\right)}_{R}^{-1}}(b,:)\right)}^{T}\;=\left(U_{A_{1}A_{2}}PM_{P_{L}^{\prime }}(s,:)\right){\left(V_{A_{k+1}A_{k+2}}PM_{{\left(P\prime \right)}_{R}^{-1}}(t,:)\right)}^{T}\;=PM_{P_{L}}(s,:){\left(PM_{P_{R}^{-1}}(t,:)\right)}^{T},$$

which establishes the result. □

Finally, the HeteSim score is given by the cosine of the angle *θ* defined by vectors $PM_{P_{L}}(s,:)$ and $PM_{P_{R}^{-1}}(t,:)$.

**Definition 8**. *The normalized HeteSim score between two objects a and b based on the relevance path P is*

$$HS(s,t|P)=cos(\theta )=\frac{PM_{P_{L}}(s,:){\left(PM_{P_{R}^{-1}}(t,:)\right)}^{T}}{\left|PM_{P_{L}}(s,:)\right|\left|{\left(PM_{P_{R}^{-1}}(t,:)\right)}^{T}\right|}.$$

**Figure 1.**
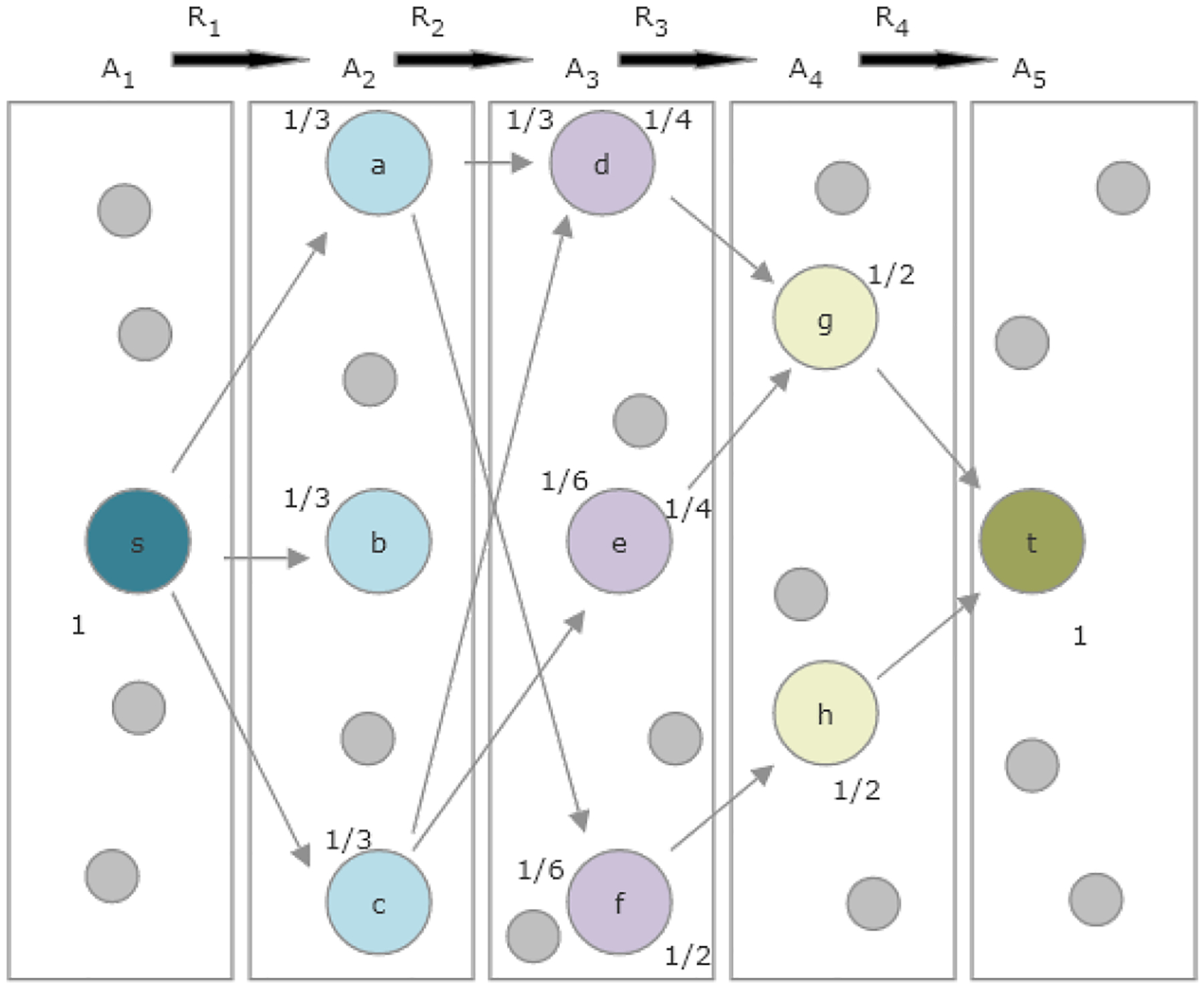
Example graph, metapath, and HeteSim computation.

The above definition uses the multiplication of transition matrices to obtain reachable probability matrices, which in turn give the HeteSim score with respect to a given metapath. We can recast this matrix multiplication in the language of random walks. Consider the example graph and metapath given in Figure 1. Beginning with node *s*, we assign the probability value 1, since this is the specified source node. Next, we distribute that probability among all neighbors of *s* with type *A*_2_ joined by an edge of type *R*_1_. These neighbors are *a*, *b* and *c*, and each of these three nodes gets labeled with the probability 1/3. We repeat the same process with the neighbors of *a*, *b*, *c* having type *A*_3_ and joined by an edge of type *R*_2_. The probability 1/3 assigned to node *a* is split between its neighbors *d* and *f*, with each neighbor receiving 1/6. Node *b* has no eligible neighbors, and so its probability mass does not propagate to the next layer of the graph. Node *c* splits its probability mass of 1/3 between *d* and *e*. Therefore, *d* is labeled with probability mass 1/3, with 1/6 coming from *a* and 1/6 from *c*. Node *e* only receives probability mass from *c* and is therefore labeled with 1/6. Similarly, node *f* receives probability mass only from *a*, and therefore has total probability mass 1/6. This computation, which is equivalent to the matrix multiplication described above, gives

$$PM_{P_{L}}(s,:)=\left[\begin{array}{l} 1/3\\ 1/6\\ 1/6 \end{array}\right].$$

To obtain $PM_{P_{R}^{-1}}(t:)$, we repeat the same procedure on the second half of the metapath, this time working backwards towards *A*_3_ from *t*. To start, *t* gets probability mass label 1. That probability is split among its 2 neighbors in *A*_4_, giving *g* and *h* each probability mass 1/2. The mass of *g* is split evenly among *d* and *e*, so both of these nodes have probability mass 1/4. All of the probability mass of *h* goes to *f*, giving *f* a probability mass 1/2. Note that we have now labeled nodes *d*, *e* and *f* twice, once from the left and once from the right. While the labels from the left gave us $PM_{P_{L}}(s:)$, the labels from the right give

$$PM_{P_{R}^{-1}}(t,:)=\left[\begin{array}{l} 1/4\\ 1/4\\ 1/2 \end{array}\right].$$

Finally, we can compute

$$HS(s,t|P)=\frac{PM_{P_{L}}(s,:){\left(PM_{P_{R}^{-1}}(t,:)\right)}^{T}}{\left|PM_{P_{L}}(s,:)\right|\left|{\left(PM_{P_{R}^{-1}}(t,:)\right)}^{T}\right|}=\frac{1/4}{1/2\cdot \sqrt{6}/4}=\frac{\sqrt{6}}{3}.$$

### 1.6. Overview of SemNet’s Existing HeteSim Implementation

The implementation of HeteSim in SemNet version 1 includes more than just the single-metapath HeteSim computation described in Section 1.5. In SemNet, HeteSim is not just used to give a score of the relatedness of two specific nodes with respect to a fixed metapath. Instead, it is used as a tool to rank a set of candidate source nodes based on their relatedness to a fixed target node.

**Figure 2.**
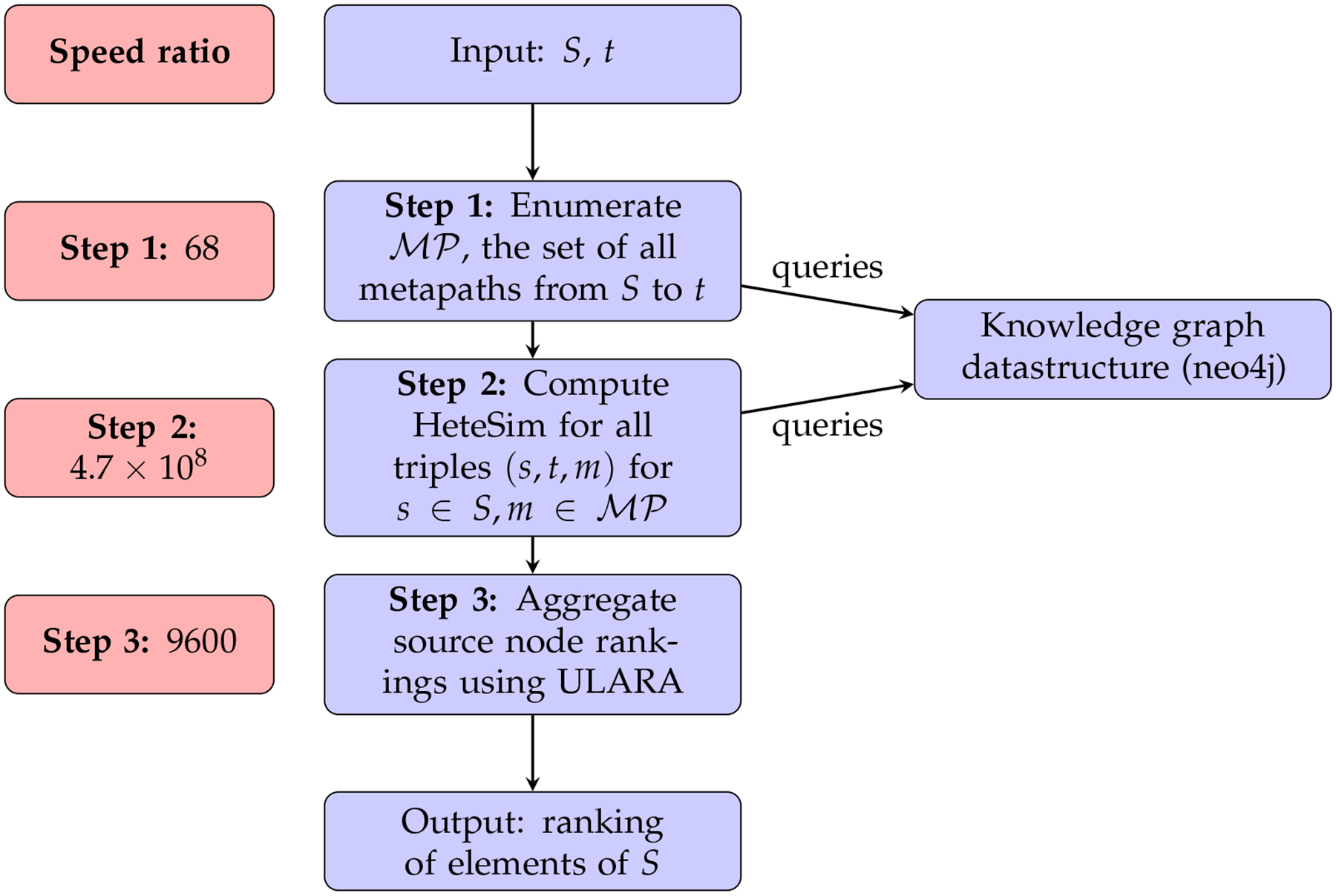
Overview of SemNet version 1 HeteSim implementation. Speed ratio is computed as (SemNet 1 time)/(SemNet 2 time) and is given for source node insulin and target node Alzheimer’s disease. In SemNet 2, the approximate mean HeteSim algorithm is used with approximation parameters *ϵ* = 0.1 and *r* = 0.9.

Figure 2 gives an overview of this ranking algorithm as it exists in SemNet version 1. As input, the algorithm accepts a set of candidate source nodes *S* and a single target node *t*. In step 1, the set of all metapaths MP which have an instance joining some element of *S* to *t* is enumerated. This enumeration depends upon the underlying knowledge graph, which is stored in Neo4j. Step 2 is the computation of HeteSim scores for each triple (*s*, *t*, *m*) for *s* ∈ *S*, m∈MP. For any fixed metapath m∈MP, the results from step 2 induce a ranking on the source nodes *S* by HeteSim score. Step 3 takes these |MP| rankings and combines them to form a single ranking using a technique called ULARA (see [14]). Finally, this combined ranking is returned to the user and is used as an indication of which nodes from *S* are most closely related to *t*.

In this work, we will keep the overall structure of the HeteSim algorithm outlined in Figure 2, but will make several substantial changes to the various subroutines. First, we will replace the knowledge graph data structure using Neo4j with one based solely on Python dictionaries. Second, we will explore algorithms using randomization as candidate replacements for Step 2. Finally, we will discuss a flaw in ULARA and will replace Step 3 with the generation of a ranking based on mean HeteSim score over all metapaths. We will also explore an approximate version of Step 3 where only a subset of metapaths are selected for inclusion in the mean.

## 2. Methods

### 2.1. A New Method for Combining HeteSim Scores from Multiple Metapaths

SemNet version 1 outputs a ranking of many candidate source nodes with respect to a fixed target node. This ranking is intended to reflect the overall relatedness of each source node to the target node. SemNet version 1 computes the HeteSim scores for all requested source nodes and for all possible metapaths (up to some length bound) joining those source nodes to the target node. Each metapath induces a ranking of the source nodes according to HeteSim score. In order to combine these many rankings into a single ranking, SemNet version 1 uses a technique called ULARA (Unsupervised Learning Algorithm for Rank Aggregation) [14]. Due to a flaw in ULARA, this work replaces ULARA with a ranking based on mean HeteSim scores.

#### 2.1.1. Background on ULARA

ULARA (Unsupervised Learning Algorithm for Rank Aggregation) [14] was developed by Klevmetiev et al. to solve the problem of *rank aggregation*. Rank aggregation considers the question of how to combine multiple rankings of a set of objects. Consider, for example, the problem of combining the results of multiple search engines into a single “best” ranking. Each search engine gives a different ordering of results. When the search engines disagree on which items are more relevant than other items, it is not immediately clear how to resolve this discrepancy and output a “best” ordered list of search results. ULARA proposes one solution to this problem based on an optimization problem. Conceptually, ULARA computes with mean rank of each object. The algorithm then finds a linear combination of the input ranking functions, giving more weight to functions that agree more closely with the mean ranking.

We now move to a formal mathematical exposition of ULARA. Note that we explain ULARA in the full generality with which it is presented in [14], but SemNet version 1 does not require the full generality of ULARA and may be thought of as using a special case.

Let *X* be a set of objects to be ranked, and let *Q* be a set of valid queries. Let *x*, *x*′ ∈ *X*, *q* ∈ *Q*. Let r:Q×X→ℕ be a ranking function, so that *r*(*q*, *x*) < *r*(*q*, *x*′) means that *x* has a higher ranking than *x*′ with respect to the query *q*. Let N∈ℕ. Given a set of ranking functions ${\left\{r_{i}\right\}}_{i=1}^{N}$, ULARA produces a ranking function of the form

$$R(q,x)=\overset{N}{\underset{i=1}{\sum }}w_{i}r_{i}(q,x),$$

for some real numbers ${\left\{w_{i}\right\}}_{i=1}^{N}$ satisfying 0 ≤ *w*_*i*_ ≤ 1 for all 1 ≤ *i* ≤ *N* and $\sum_{i=1}^{N}w_{i}=1$. The value of each *w*_*i*_ is determined by an optimization problem. Let

$$\mu (q,x)=\frac{\sum_{i:r_{i}(q,x)\leq \kappa_{i}}r_{i}(q,i)}{{\left|\left\{i:r_{i}(q,x)\leq \kappa_{i}\right\}\right|}^{\prime }}$$

where *κ*_*i*_ is a threshold which allows for the possibility that not every ranking function returns a rank for every *x* ∈ *X*. The function *μ*(*q*, *x*) is intended to represent the mean ranking of element *x* with respect to query *q* over all ranking functions *r*_*i*_. Let

$$\sigma_{i}={\left(r_{i}(q,x)-\mu (q,x)\right)}^{2}.$$

This variance-like function is used to measure the agreement of ranking functions with each other, with the goal of giving ranking functions that agree with the mean a higher weight. Let

$$\delta_{i}(q,x)=w_{i}\sigma_{i}(q,x).$$

We can now finally state the optimization problem at the center of ULARA:

$$\underset{w_{1},\ldots ,w_{N}}{\text{arg min}}\underset{q\in Q}{\sum }\underset{x\in X}{\sum }\overset{N}{\underset{i=1}{\sum }}\delta_{i}(q,x),$$

subject to the constraints

$$\overset{n}{\underset{i=1}{\sum }}w_{i}=1\text{ and }\forall i,w_{i}>0.$$

Note that this optimization problem is intended assign more weight to the ranking functions that agree most closely with the average ranking. ULARA solves the optimization problem using gradient descent. The details of the gradient descent algorithm are not relevant to the conceptual flaw in ULARA and are not presented here.

#### 2.1.2. A Flaw in ULARA

The flaw in ULARA can be seen simply by examining the optimization problem itself. Let

$$a_{i}=\underset{q\in Q}{\sum }\underset{x\in X}{\sum }\sigma_{i}(q,x).$$

Then, the optimization problem becomes

$$\underset{w_{1},\ldots ,w_{N}}{\text{arg min}}\overset{N}{\underset{i=1}{\sum }}w_{i}a_{i},$$

subject to the constraints

$$\overset{N}{\underset{i=1}{\sum }}w_{i}=1\text{ and }\forall i,w_{i}>0.$$

Let *j* be such that *a*_*j*_ = min_*i*_ *a*_*i*_. Then, an optimal solution is given by

$$w_{i}=\{\begin{array}{ll} 1 & \text{if }i=j\\ 0 & \text{if }i\neq j \end{array}.$$

Further, the solution is unique if *a*_*j*_ is the unique minimum of the set *A* = {*a*_1_, …, *a*_*N*_}. The case where the optimization problem does not have a unique solution is not mentioned in [14], and it seems this case should be rare in practice. Therefore, any unique optimal solution of the ULARA optimization problem places all of the available weight on a single ranking function. That is, ULARA does not give an aggregation of ranking functions; it simply selects a single raking function which shows most agreement with the others. In the language of SemNet, this should mean that only one metapath is used to give the final ranking of source nodes.

#### 2.1.3. Implications for SemNet

Despite the fact that the math shows that only one metapath should have been used to generate rankings in SemNet version 1, this is not what actually happened. If only 1 metapath had actually been used to compute the rankings for SemNet version 1, it would be seemingly impossible that the produced ranking results would make sense. Yet, in multiple cases examined by domain experts in various fields (Alzheimer’s disease, amyotrophic lateral sclerosis, leukemia, SARS coronavirus, and many more), the SemNet version 1 ranking results were quite intuitive. Thus, it was necessary to reconcile how the produced SemNet version 1 rankings would appear generally accurate despite the identified flaw in the original ULARA algorithm published by Klementiev and colleagues [14]. As such, a line by line examination of the adopted implementation of ULARA in the actual SemNet version 1 code [13] was performed and compared to the original published ULARA implementation [14]. The careful evaluation of the adopted ULARA implementation in SemNet version 1 identified a previously unseen but helpful coding bug that partially fixed the issue with the original ULARA. Specifically, the code in SemNet version 1 resulted in the ULARA algorithm terminating before the gradient descent had converged. As a result, a linear combination of multiple ranking functions (with nonzero coefficients) was actually returned, and multiple metapaths therefore are reflected in the rankings given by SemNet. Thus, unlike the original and above described ULARA, which would have only used 1 metapath to perform the ranking, the helpful bug in the ULARA implementation within SemNet version 1 used a partially averaged ranking that contained multiple metapaths. As such, SemNet version 1 was still able to be used by domain scientists to produce helpful and seemingly sensible rankings. While the serendipitous bug rendered SemNet version 1 useful, a fundamentally correct replacement for the ULARA algorithm is necessary.

As a replacement for ULARA, in SemNet version 2, the mean HeteSim score of a source node with respect to all metapaths is used to generate a ranking of source nodes.

### 2.2. Computational Analysis of HeteSim Runtimes: SemNet Version 1

To better understand the runtime of the HeteSim computation, the Python module time [36] was used to record the time required to compute HeteSim for each of the metapaths from the studied source nodes to Alzheimer’s disease. Additionally, the total time spent on the required Neo4j queries was recorded for each metapath. This allows separate analysis of the time required to query the graph and the time required to perform the HeteSim computations.

### 2.3. Development, Implementation, and Testing of Algorithms

The core development work for this project can be divided into three general categories: re-implementation of the knowledge graph data structure, development and implementation of algorithms, and testing.

#### 2.3.1. Knowledge Graph Data Structure

SemNet version 1 used Neo4j to store the knowledge graph. After preliminary testing showed that Neo4j was likely a significant bottleneck, the knowledge graph data structure was re-implemented using nested Python dictionaries. Because these dictionaries use hashing for lookup, they have average lookup time *O*(1) (see, e.g., [37]). As a result, dictionaries allow for quickly examining the neighborhood of a node in the knowledge graph, restricted to edge and node types of interest. Consequently, it is also efficient to traverse paths within the graph.

After testing on artificial examples, a knowledge graph object was built using an edge set derived from SemMedDB. This is an updated version of the edge set, and is not identical to the edge set from SemNet version 1.

#### 2.3.2. Development of Approximation Algorithms

In addition to the data structure improvements, approximation algorithms based on randomization were explored as a way of further increasing performance. In particular, approximation algorithms were investigated as possible replacements for the computation of HeteSim on a single metapath (step 2 in Figure 2) and aggregation of rankings (step 3 in Figure 2).

#### 2.3.3. Implementation and Testing

All code were implemented in Python 3. Testing was performed using Jupyter Notebook 5.5.0 [38] and Python 3.6.10 [39]. All code were run on a server with 1 NVIDIA TESLA v100 GPU with 32 GB RAM and a 48 core CPU with 320 GB RAM.

For all code not involving randomization, the correctness of implementation was assessed using unit tests, which may be found in the source code repository. The one randomized function of significant complexity, randomized pruned HeteSim, was assessed on artificially-constructed example knowledge graphs. These examples were constructed by hand by the authors, and the full examples may be found in the source code repository. The algorithm was run on each graph 100 times with parameters *ϵ* = 0.05 and *r* = 0.95. As with the SemNet version 1 implementation, the speed of the new implementation was assessed using the Python time module [36].

#### 2.3.4. User Study Methods

A small user study was performed to quantify the significant differences between two groups of users: a group of naive SemNet version 1 users (n = 11) and a group of naive SemNet version 2 users (n = 10) to determine how many users were comfortable in running a simulation after a short standardized training session that also included reading the user documentation. To ensure degree of previous Python experience was not biasing the analysis, groups were selected to ensure equivalent distributions of prior Python user experience. Additionally, a third group of users (n = 7) trained in both SemNet version 1 and SemNet version 2 was used to compare the user friendliness of SemNet version 1 and version 2. A simple categorical standardized electronic survey was used to quantify comfort in using SemNet version 2 and its user friendliness. Details are provided in the Results in Section 3.4. Fisher’s exact test was used to perform statistical analysis in Microsoft Excel.

## 3. Results

### 3.1. Computational Analysis of HeteSim Runtimes: SemNet Version 1

**Figure 3.**
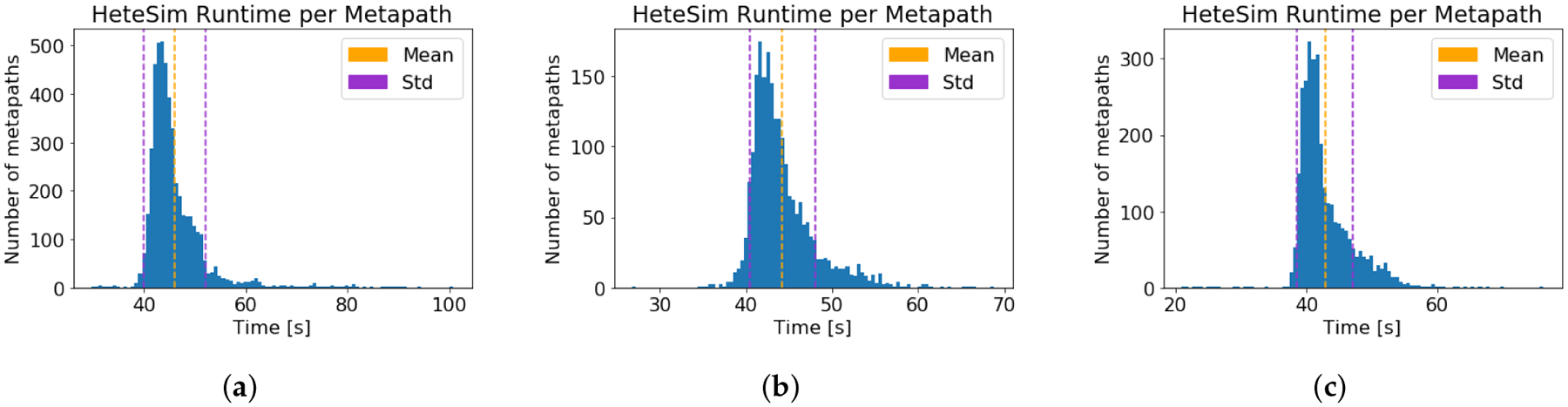
Distribution of SemNet version 1 HeteSim computation times for all metapaths joining the given source node and Alzheimer’s disease. (**a**) Insulin; (**b**) Hypothyroidism; (**c**) Amyloid.

**Figure 4.**
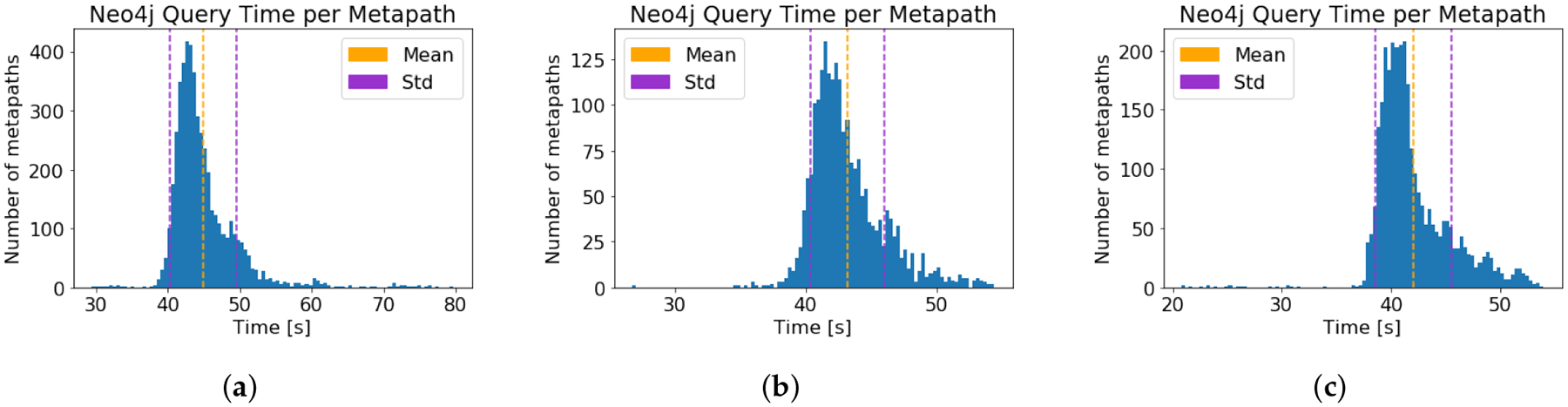
Distribution of Neo4j query times in SemNet version 1 HeteSim computation for all metapaths joining the given source node and Alzheimer’s disease. (**a**) Insulin; (**b**) Hypothyroidism; (**c**) Amyloid.

For each of the three source nodes, the runtime of the HeteSim computation on each metapath from the source node to Alzheimer’s disease was recorded. The computation time results are given in Table 1, and the distribution of runtimes is depicted graphically in Figure 3. Note that SemNet version 1 incorporated parallelization, allowing multiple HeteSim computations for different metapaths to occur simultaneously. Therefore, the computation time per metapath times the number of metapaths does not equal the total computation time. Time required for the neo4j graph queries was also measured and is displayed in Figure 4.

### 3.2. Algorithms

In this section, we present several algorithms for computing HeteSim and variants. Proofs of correctness are also given where appropriate.

**Figure 5.**
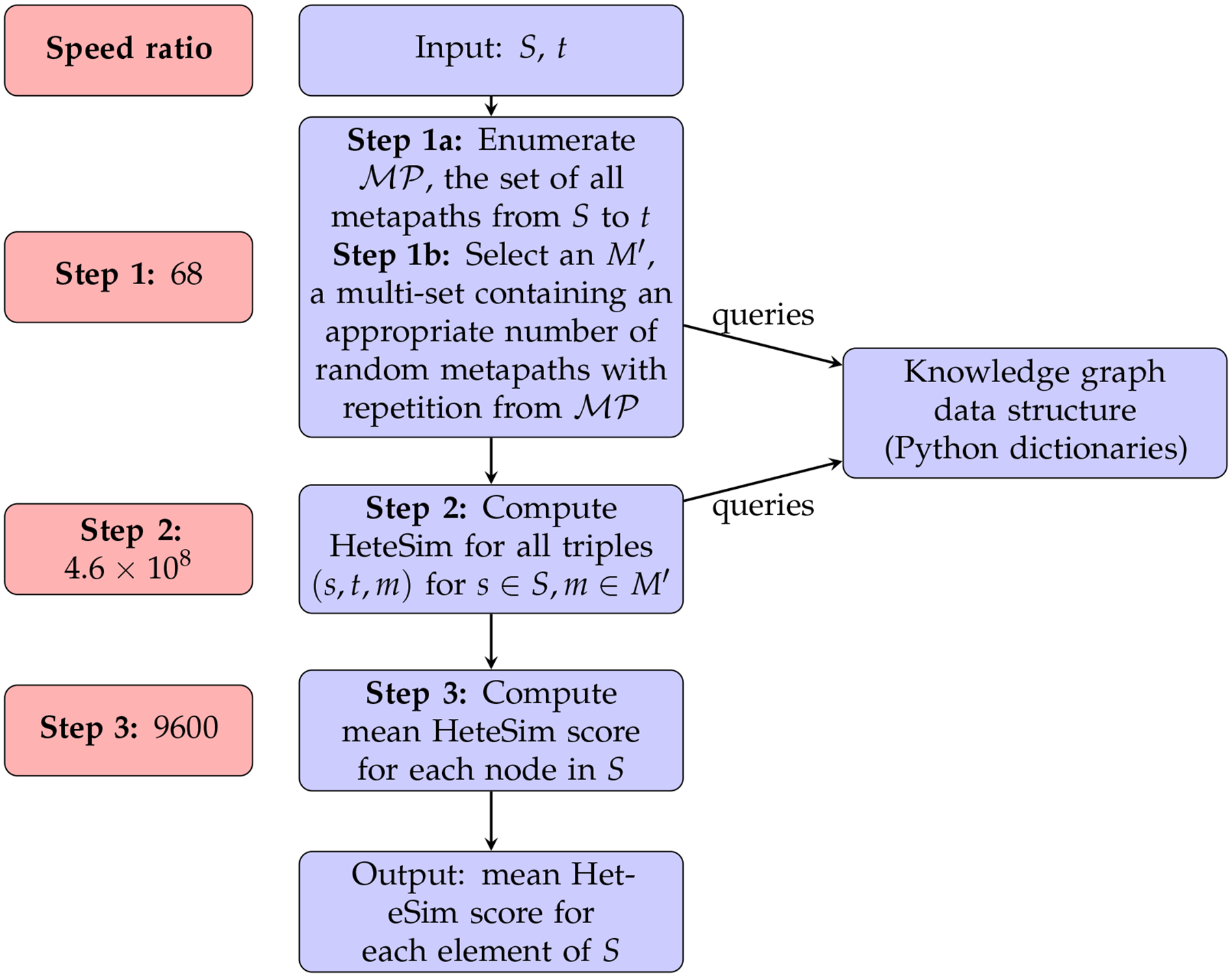
Overview of SemNet version 2 approximate mean HeteSim implementation. Speed ratio is (SemNet 1 time)/(SemNet 2 time) and is given for source node insulin and target node Alzheimer’s disease. SemNet version 2 used approximation parameters *ϵ* = 0.1 and *r* = 0.9.

We consider two main algorithms for computing HeteSim on a single metapath and two algorithms for aggregating HeteSim scores across multiple metapaths. For computing HeteSim on a single metapath, we consider the deterministic HeteSim algorithm used in SemNet version 1 and a new algorithm, randomized pruned HeteSim. For aggregating HeteSim scores over multiple metapaths, we consider computing the exact mean over all metapaths and also an algorithm which approximates the mean by taking the mean over a random subset of metapaths. We also combine these algorithms to obtain three distinct algorithms for computing (an approximation to) the mean HeteSim score: deterministic HeteSim with exact mean, deterministic HeteSim with approximate mean, and randomized pruned HeteSim with approximate mean. Using approximate mean HeteSim as an example, an overview of the new algorithm structure, emphasizing changes, is shown in Figure 5.

#### 3.2.1. Deterministic HeteSim

For completeness, we summarize the deterministic algorithm for computing HeteSim. While this same algorithm is used in SemNet version 1, SemNet version 2 significantly improves the implementation by changing the underlying data structure for the knowledge graph. Where version 1 used Neo4j, version 2 uses a knowledge graph object built from Python dictionaries.

Given a source node *s*, a target node *t*, and a metapath P, the deterministic HeteSim algorithm begins by splitting P into two halves: $P_{L}$ and $P_{R}$. If P has odd length, the construction described in Section 1.5 is applied before constructing $P_{L}$ and $P_{R}$. An identical subroutine is now applied to both $P_{L}$ and $P_{R}^{-1}$. The following exposition will consider only $P_{L}$.

Recall that the algorithm must compute $PM_{P_{L}}(s,:)$, which may be understood as the probability that a random walk along the given metapath starting from *s* arrives at a given node in *A*_*l*/2_. The algorithm iteratively computes the probability of arriving at each node in *A*_*i*_ for step *i* of the metapath for 1 ≤ *i* ≤ *l*/2.

Let *v*_*i*_(*x*) be the probability of arriving at node *x* of type *A*_*i*_ at step *i* of the metapath. To compute *v*_*i*_ for *i* > 1, note that it is sufficient to know *v*_*i*−1_, as

$$v_{i}(x)=\underset{y\in \delta_{R_{i-1}}^{-}(x)}{\sum }\frac{1}{\delta_{R_{i-1}}^{+}(y)}v_{i-1}(y).$$

Therefore, beginning with *v*_1_(*s*) = 1, the algorithm iteratively computes *v*_2_, …, *v*_*l*/2_ and $PM_{P_{L}}=v_{l/2}$. After completing the analogous computation for $P_{R}^{-1}$, the algorithm returns

$$\frac{PM_{P_{L}}(a,:){\left(PM_{P_{R}^{-1}}(b,:)\right)}^{T}}{\left|PM_{P_{L}}(a,:)\right|\left|{\left(PM_{P_{R}^{-1}}(b,:)\right)}^{T}\right|}.$$

Pseudocode is given in Algorithms 1 and 2.


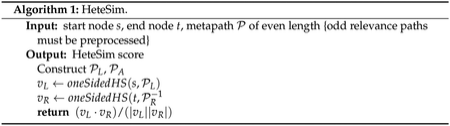



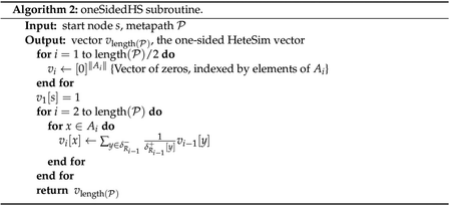


#### 3.2.2. Pruning the Graph

Given a metapath $P_{L}=A_{1}\overset{R_{1}}{\to }A_{2}\overset{R_{2}}{\to }\ldots \overset{R_{\frac{l}{2}-1}}{\to }A_{\frac{l}{2}}$, a random walk starting from 2 *s* ∈ *A*_1_ may arrive at node *u* ∈ *A*_*i*_ such that the out degree of *u* along edges of type *R*_*i*_ is 0. Informally speaking, the random walk has reached a dead end. As an example, node *b* in Figure 1 is a dead end. The presence of these dead ends reduces the probability that a random walk starting from *s* actually reaches any node of type $A_{\frac{l}{2}}$. In fact, we can construct graphs that make this probability arbitrarily small. Therefore, a basic random walk algorithm may have arbitrarily long runtime. We will address this limitation by defining a new but closely related quantity: pruned HeteSim.

**Figure 6.**
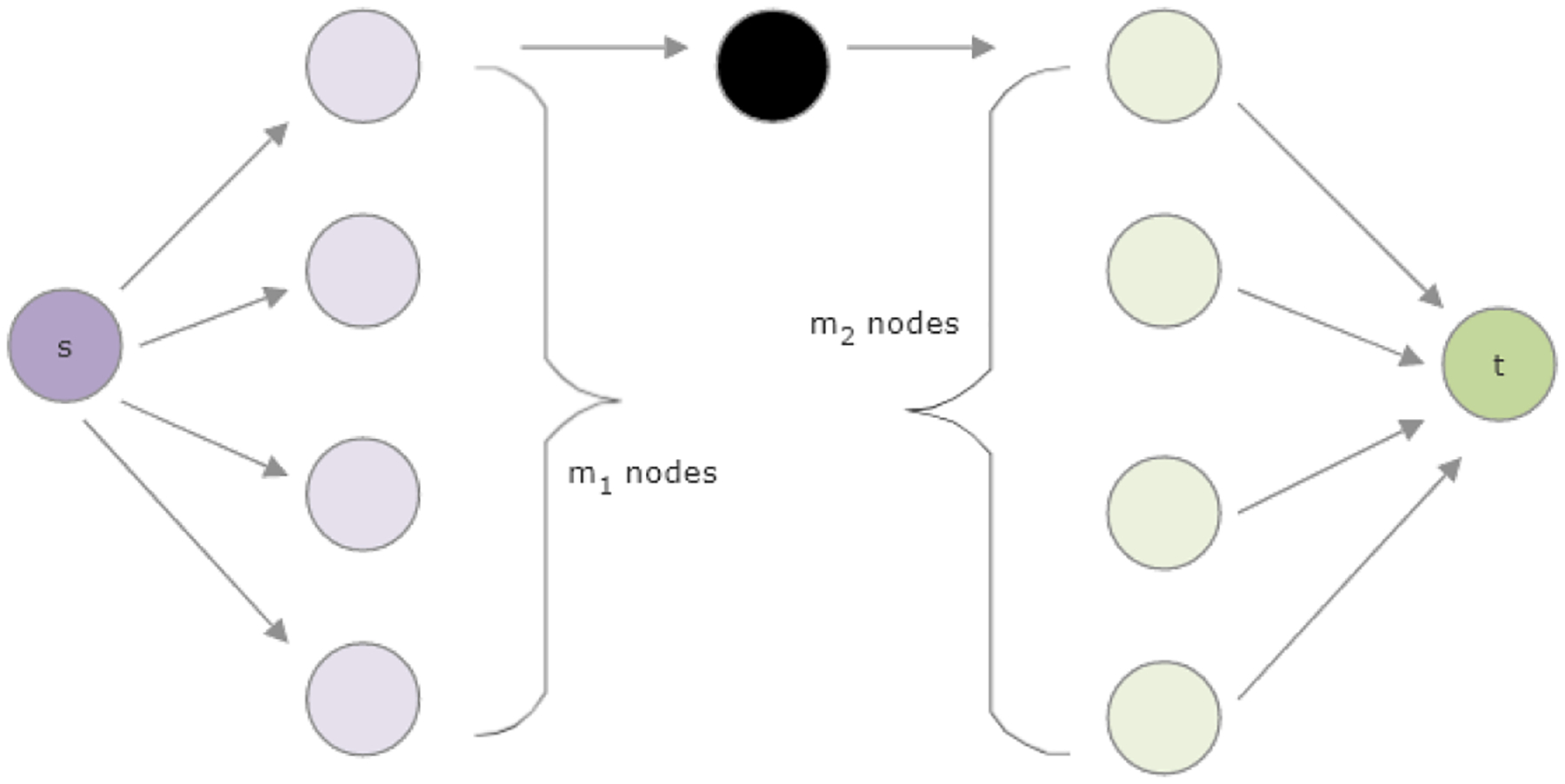
An example knowledge graph. Here, we use the convention that nodes are organized by type into vertical columns in the order that they appear in the metapath. We also only show edges that may appear in some metapath instance. This example has *m*_1_ − 1 dead-end nodes on the left and *m*_2_ − 1 dead-end nodes on the right. The HeteSim score of *s* and *t* with respect to the metapath is 1 for all values of *m*_1_ and *m*_2_.

Before proceeding, we provide two additional examples to explore the effect of dead ends on HeteSim scores. In Figure 6, a simple knowledge graph is shown, organized according to one metapath. The nodes are organized into columns by type, and the columns are given in the order that those types appear in the metapath. The only edges shown are those which appear in some instance of the metapath. This graph has *m*_1_ − 1 dead-end nodes on the left-hand side and *m*_2_ − 1 dead-end nodes on the right-hand side. We can compute its HeteSim score as follows.


$$\text{HS}(s,t|P)=\frac{1\cdot 1}{1\cdot 1}=1.$$

Note that this score does not change with *m*_1_ or *m*_2_. In particular, the HeteSim score with the given graph is identical to the HeteSim score when all dead ends are removed from the graph. As we will later see, this result generalizes to all metapaths of length less than or equal to 4.

**Figure 7.**
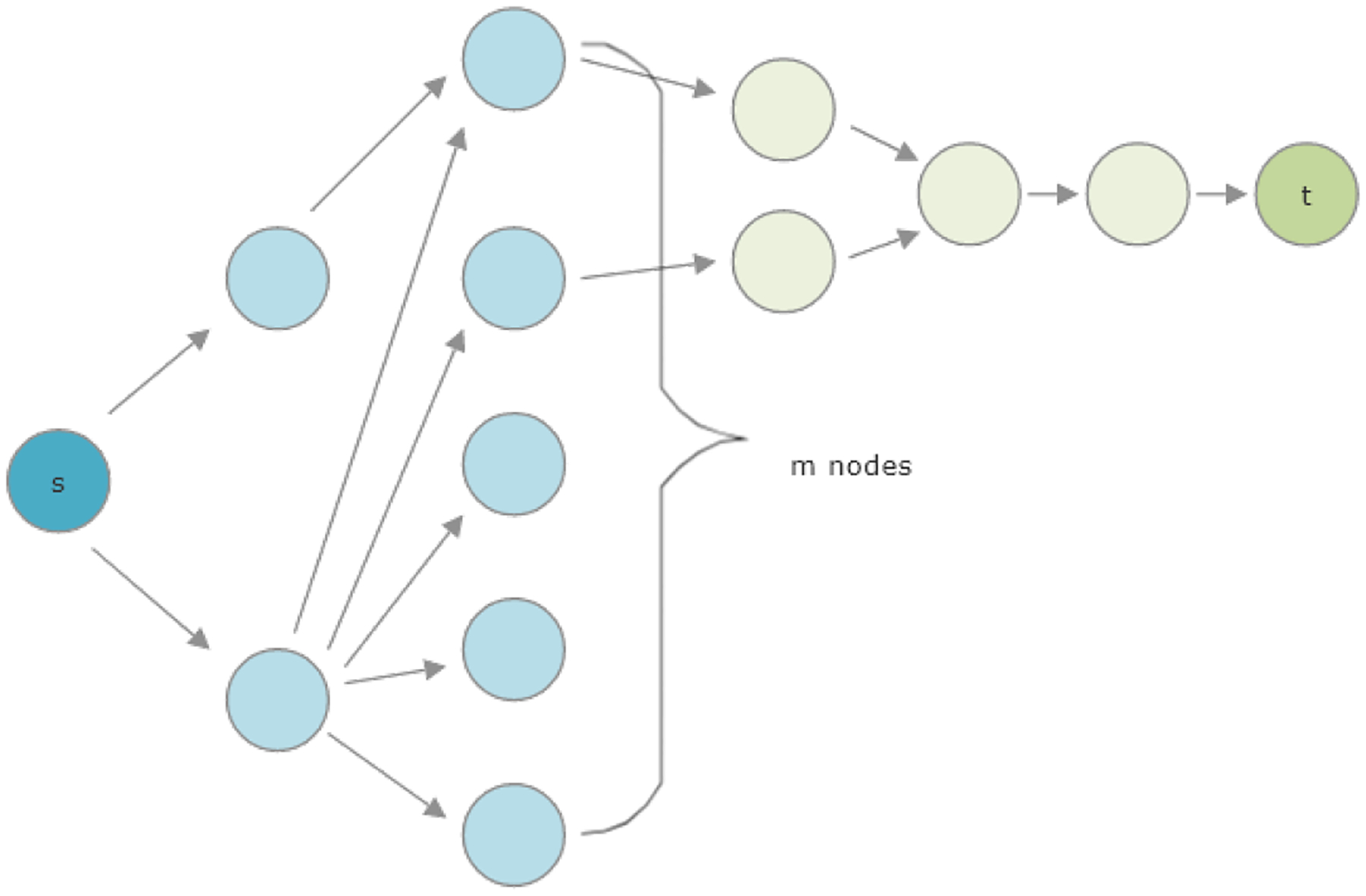
An example metapath and knowledge graph, drawn with the same conventions as in Figure 6. Note that, in this example, the removal of dead ends does change the HeteSim score.

In contrast, the metapath and knowledge graph depicted in Figure 7 create a situation where the removal of dead ends does change the HeteSim score. If we take *m* = 2, then we have removed all dead-end nodes. In this case, the HeteSim score is

$$\text{HS}(s,t|P)=\frac{\left[\begin{array}{cc} 3/4 & 1/4 \end{array}\right]{\left(\left[\begin{array}{ll} 1/2 & 1/2 \end{array}\right]\right)}^{T}}{\left|\left[\begin{array}{c} \begin{array}{ll} 3/4 & 1/4 \end{array} \end{array}\right]\right|\left|\left[\begin{array}{ll} 1/2 & 1/2 \end{array}\right]\right|}=\frac{1/2}{\sqrt{5/8}\sqrt{1/2}}=\frac{2\sqrt{5}}{5}.$$

If we instead take *m* = 3, then the HeteSim score is $\frac{5\sqrt{34}}{34}$, and, in the limit as *m* → ∞, the HeteSim score approaches $\frac{\sqrt{2}}{2}$.

We now introduce a new score: Pruned HeteSim. This new score is identical to HeteSim on relevance paths of length at most 4. To rigorously define Pruned HeteSim, we must first formally define a dead-end node at step *i* of a given metapath and with respect to nodes *s* and *t*.

Let *G* = (*V*, *E*) be a heterogeneous information network, and let $P=A_{1}\overset{R_{1}}{\to }A_{2}\overset{R_{2}}{\to }\ldots \overset{R_{l}}{\to }A_{l+1}$ be a metapath in *G*. Let *s* ∈ *V* with *ψ*(*s*) = *A*_1_ and *t* ∈ *V* with *ψ*(*t*) = *A*_*l*_. Let *C*_1_ be the set of nodes of type *A*_*l*/2_ reachable from *s* along metapath $P_{L}$. Similarly, let *C*_2_ be the set of nodes of type *A*_*l*/2_ reachable from *t* along metapath $P_{R}^{-1}$. Let *C* = *C*_1_ ∩ *C*_2_, and label the elements of *C* so that *C* = {*c*_1_, *c*_2_, …, *c*_*j*_}. For *i* ≤ *j*, let *X*_*i*_ be the event that a random walk starting at *s* along $P_{L}$ ends at node *c*_*i*_. Similarly, let *Y*_*i*_ be the event that a random walk starting at *t* along $P_{R}^{-1}$ ends at node *c*_*i*_. Let *x*_*i*_ = *Pr*(*X*_*i*_) and *y*_*i*_ = *Pr*(*Y*_*i*_). Let *x* = (*x*_1_, *x*_2_, …, *x*_*j*_) and let *y* = (*y*_1_, *y*_2_, …, *y*_*j*_).

Let *Z* be the event that a random walk starting from *s* along $P_{L}$ reaches some node in *C*. Similarly, let *W* be the event that a random walk starting from *t* along $P_{R}^{-1}$ reaches some node in *C*.

**Definition 9**. *For a node v belonging to any of A*_1_, *A*_2_, …, *A*_l/2_*, we define a dead end as follows*. *Let metapath* P *and source node s be fixed*. *Let A be the event that a random walk beginning from s and following metapath* $P_{L}$ *contains node v at step i (so that the type of v is A*_*i*_*)*. *Then, v is a* dead end *at step i of metapath P and with respect to source node s if and only if Pr*(*Z*|*A*) = 0. *For a node w belonging to any of A*_*l*/2+1_, …, *A*_*l*+1_*, the definition is analogous*. *Let metapath P and target node t be fixed*. *Let B be the event that a random walk starting from t and following metapath $P_{R}^{-1}$ contains node w at step i*. *Then, w is a* dead end *with respect to step i of metapath, P and target node t if and only if Pr*(*W*|*B*) = 0. *For fixed nodes s*, *t and fixed metapath P, let D*_*i*_ *be the set of dead-end nodes at step i of metapath P with respect to source node s and target node t*.

Informally, this definition means that a node *v* is a dead end at step *i* of a metapath if no random walk which reaches the set of central nodes *C* has *v* as its *i*th node. Recall that non-normalized HeteSim is defined by

$$h\left(s,t|R_{1}\circ R_{2}\circ \cdots \circ R_{l}\right)=\frac{1}{\left|O\left(s|R_{1}\right)\right|\left|I\left(t|R_{l}\right)\right|}\underset{a\in O\left(s|R_{1}\right)}{\sum }\underset{b\in I\left(t|R_{l}\right)}{\sum }h\left(a,b|R_{2}\circ R_{3}\circ \cdots \circ R_{l-1}\right),$$

where *O*(*s*|*R*_1_) is the set of out-neighbors of node *s* based on relation *R*_1_, and *I*(*t*|*R*_*l*_) is the set of in-neighbors of node *t* based on the relation *R*_*l*_. To define the non-normalized version of pruned, we simply exclude dead-end nodes from the sets of neighbors.

**Definition 10**. *Let $P=R_{1}\circ R_{2}\circ \cdots \circ R_{l}$ be a metapath in some graph G*. *Let s*, *t belong to the vertex set of G, and let D*_*i*_ *be the set of dead-end nodes at step i of metapath P*. *Then, the non-normalized pruned HeteSim score is given by*

$$g\left(s,t|R_{1}\circ R_{2}\circ \cdots \circ R_{l}\right)=\frac{1}{\left|O\left(s|R_{1}\right)\setminus D_{1}\right|\left|I\left(t|R_{l}\right)\setminus D_{l}\right|}\underset{a\in O\left(s|R_{1}\right)\setminus D_{1}}{\sum }\underset{b\in I\left(t|R_{l}\right)\setminus D_{l}}{\sum }h\left(a,b|R_{2}\circ R_{3}\circ \cdots \circ R_{l-1}\right),$$

*where O*(*s*|*R*_1_) *is the set of out-neighbors of node s based on relation R*_1_*, and I*(*t*|*R*_*l*_) *is the set of in-neighbors of node t based on the relation R*_*l*_.

The normalization of pruned HeteSim proceeds exactly like that for HeteSim. We obtain a restricted adjacency matrix $W_{AB,i}^{\prime }$ for the relation $A\overset{R_{i}}{\to }B$ by removing any 1s in *W*_*AB*_ corresponding to a dead-end node in *B* at step *i* of the metapath. As before, we normalize $W_{AB,i}^{\prime }$ along its row vectors to obtain $U_{AB,i}^{\prime }$. As before, we can obtain a reachable probability matrix by multiplying the normalized restricted adjacency matrices:

$$PM_{P}^{\prime }=U_{A_{1}A_{2},2}^{\prime }U_{A_{2}A_{3},3}^{\prime }\ldots U_{A_{l}A_{l+1},l+1}^{\prime }.$$

**Definition 11**. *The normalized pruned HeteSim score is given by*

$$PHS(a,b|P)=\frac{PM_{P_{L}}^{\prime }(a,:){\left(PM_{P_{R}^{-1}}^{\prime }(b,:)\right)}^{T}}{\sqrt{\left|PM_{P_{L}}^{\prime }(a,:)\right|\left|{\left(PM_{P_{R}^{-1}}^{\prime }(b,:)\right)}^{T}\right|}}.$$

Note that, for metapaths with no repeated node types, pruned HeteSim may be computed by simply removing all dead-end nodes from the graph and then computing HeteSim on this pruned graph. Importantly, pruned HeteSim has value equal to plain HeteSim for metapaths of length at most 4. Since these shorter paths are often the ones of most interest in small-diameter knowledge graphs, pruned HeteSim may be thought of as a replacement for HeteSim in these circumstances.

Additionally, note that Definition 11 gives rise to a deterministic algorithm for computing pruned HeteSim, much like the deterministic algorithm for HeteSim. The algorithm now requires 2 passes over the data structure. In the first pass over the data, dead ends are identified. In a second pass, Definition 11 allows for the computation of the non-normalized pruned HeteSim score. Normalization is applied as the final step. Because our computational focus in this manuscript is on short paths of length at most four, and because HeteSim and pruned HeteSim have the same values for paths of length at most four, we do not pursue the deterministic algorithm for pruned HeteSim further. For these short paths, a deterministic computation of HeteSim is faster than a deterministic computation of pruned HeteSim.

**Theorem 1**. *Let $P=A_{1}\overset{R_{1}}{\to }A_{2}\overset{R_{2}}{\to }\ldots \overset{R_{l}}{\to }A_{l+1}$ be a metapath with length l* ≤ 4. *Then*,

PHS(s,t∣G,P)=HS(s,t∣G,P).

**Proof**. First, note that we only need to consider metapaths with even length, as odd metapaths will simply be transformed to even length metapaths before HeteSim is computed. Next, note that the result is trivial for metapaths with length 2, as these can have no dead ends. We may therefore focus only on the case where the metapath has length 4.

Let $P=A_{1}\overset{R_{1}}{\to }A_{2}\overset{R_{2}}{\to }A_{3}\overset{R_{3}}{\to }A_{4}\overset{R_{4}}{\to }A_{5}$ be a metapath in *G*. Note that there can be no dead ends of type *A*_3_. Additionally, if *s* or *t* is a dead end, then HS(s,t∣G,P)=0=PHS(s,t∣G,P). Therefore, we may assume that all dead ends are of type *A*_2_ or *A*_4_.

Recall that *X*_*i*_ is the event that a random walk in *G* from *s* reaches node *c*_*i*_, and similarly *Y*_*i*_ is the event that a random walk in *G* starting at *t* arrives at node *c*_*i*_. Let $X_{i}^{\prime }$ be the event that a random walk in *G*′ along metapath $P_{L}$ starting from *s* arrives at node *c*_*i*_. Similarly let $Y_{i}^{\prime }$ be the event that a random walk in *G*′ along metapath $P_{R}^{-1}$ arrives at node *c*_*i*_. Let *p*_*L*_ be the probability that a random walk starting from *s* arrives at a dead-end node in *A*_2_. Similarly, let *p*_*R*_ be the probability that a random walk beginning at *t* will arrive at a dead end in *A*_4_. Note that, once a random walk has reached a non-dead-end node of type *A*_2_ or *A*_4_, that random walk must reach some node of type *A*_3_. Therefore,

$$Pr\left(X_{i}\right)=\left(1-p_{L}\right)Pr\left(X_{i}^{\prime }\right)$$

and

$$Pr\left(Y_{i}\right)=\left(1-p_{R}\right)Pr\left(Y_{i}^{\prime }\right).$$

Letting *x*_*i*_ = *Pr*(*X*_*i*_), *y*_*i*_ = *Pr*(*Y*_*i*_), $x_{i}^{\prime }=Pr\left(X_{i}^{\prime }\right)$, and $y_{i}^{\prime }=Pr\left(Y_{i}^{\prime }\right)$, observe

$$\text{HS}(s,t|G,P)=\frac{\sum_{i=1}^{k}x_{i}y_{i}}{\sqrt{\sum_{i=1}^{k}x_{i}^{2}\sum_{i=1}^{k}y_{i}^{2}}}\;=\frac{\sum_{i=1}^{k}\left(1-p_{L}\right)x_{i}^{\prime }\left(1-p_{R}\right)y_{i}^{\prime }}{\sqrt{\sum_{i=1}^{k}{\left(1-p_{L}\right)}^{2}{\left(x_{i}^{\prime }\right)}^{2}\sum_{i=1}^{k}{\left(1-p_{R}\right)}^{2}{\left(y_{i}^{\prime }\right)}^{2}}}\;=\frac{\sum_{i=1}^{k}x_{i}^{\prime }y_{i}^{\prime }}{\sqrt{\sum_{i=1}^{k}{\left(x_{i}^{\prime }\right)}^{2}\sum_{i=1}^{k}{\left(y_{i}^{\prime }\right)}^{2}}}\;=\text{PHS}(s,t|G,P).$$

□

#### 3.2.3. Pruned HeteSim

We now present an alternate algorithm for computing a variant of the HeteSim score. This algorithm is much more computationally tractable, and we have shown that the HeteSim and pruned HeteSim scores are identical for relevance paths of length at most 4.

Let P be a metapath, and let *s* and *t* be source and target notes, respectively. Let *N* be a positive integer, the required value of which will be determined later. Starting from *s* the algorithm takes *N* random walks along $P_{R}$, never visiting any node that has been marked as a dead end for the current step of the metapath. At any point, if the algorithm encounters a dead end, it marks the current node as a dead end for the current step of the metapath and then retraces its steps until a non-dead-end node is reached, marking dead ends along the way as necessary. Note that any dead end at a given step in the metapath will only need to be marked once, and the algorithm will avoid it for all future random walks. The same algorithm is repeated along metapath $P_{L}^{-1}$ starting from *t*.

The frequency vectors of the terminal nodes of the random walks give an approximation for $PM_{P_{L}}^{\prime }$ and $PM_{P_{R}^{-1}}^{\prime }$, which are used to approximate the pruned HeteSim score. Psuedocode is given in Algorithms 3–5. Analysis of the algorithm, determination of *N*, and a formal proof of correctness are given in Section 3.2.4.


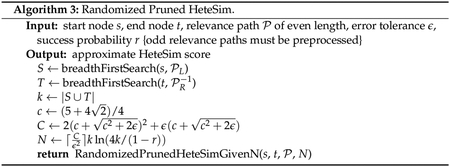



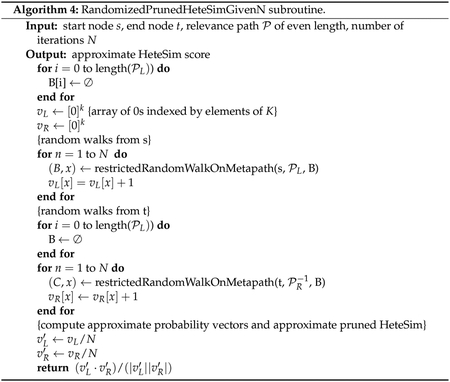



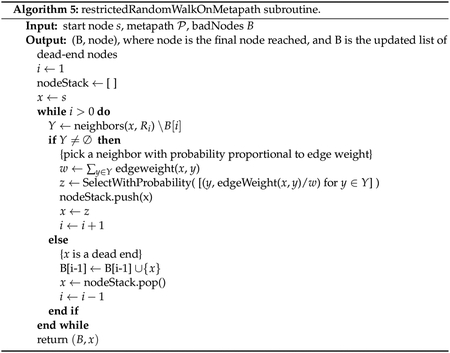


#### 3.2.4. Runtime Analysis of the Pruned HeteSim Algorithm

We now provide guarantee on the number of random walks required to approximate pruned HeteSim with a given error tolerance *ϵ* and success probability *r*.

Let $S_{k}=\left\{v\in {\mathbb{R}}^{k}:\sum_{i}v_{i}=1\text{ and }v_{i}\geq 0\right\}$. We consider arbitrary *v*, $w\in S_{k}$ for fixed *k*, where $v=PM_{P_{L}}^{\prime }(s,:)$ and $w=PM_{P_{R}^{-1}}^{\prime }(t,:)$. We will show that if all the entries in the vectors are sufficiently close to their true value, then the cosine will be sufficiently close to the true value. We consider $\hat{v}$, a random approximation of *v* after some number of steps. Notice that $\hat{v}=v+\lambda$, where $\lambda \in {\mathbb{R}}^{k}$ such that $\sum_{i=1}^{k}\lambda_{i}=0$ and *v*_*i*_ + *λ*_*i*_ ≥ 0 (since $\hat{v}$ is always a probability vector). Let

$$E_{k}(v,\delta ,\alpha ,\beta )=\left\{w\in {\mathbb{R}}^{k}:\underset{i}{\sum }w_{i}=0,v_{i}+w_{i}\geq 0,v_{i}\geq \alpha \Rightarrow \left|w_{i}\right|\leq \delta \left|v_{i}\right|,\text{ and }v_{i}<\alpha \Rightarrow \left|w_{i}\right|\leq \beta \delta \right\}.$$

We now consider $\lambda \in E_{k}(v,\delta ,\alpha ,\beta )$. Note that the bound imposed by $E_{k}(v,\delta ,\alpha ,\beta )$ treats small entries and large entries in *v* differently. This will be important to achieve an *O*(*k* log *k*) bound on the number of required random walks *N* later in the section.

We start by giving sufficient conditions for a bound on |cos *θ*′ − cos *θ*|, where *θ*′ is the angle between $\hat{v}$ and $\hat{w}$ and *θ* is the angle between *v* and *w*.

**Theorem 2**. *Fix ϵ* > 0. *Let* 0 ≤ *β*, $\bar{\beta }\leq 1$. *Let α*, $\bar{\alpha }\geq 0$. *Let v*, $w\in S_{k}$. *Let*

$$b=\frac{2+\frac{k\beta^{2}}{2|v{|}^{2}}}{1+\frac{1}{\left|v\right|\sqrt{k}}}+\sqrt{\frac{k\beta^{2}}{|v{|}^{2}}+1}$$

*and*

$$a=\frac{\frac{k\beta^{2}}{|v{|}^{2}}+1}{1+\frac{1}{\left|v\right|\sqrt{k}}}$$

*and*

$$\bar{b}=\frac{2+\frac{k{\bar{\beta }}^{2}}{2|w{|}^{2}}}{1+\frac{1}{\left|w\right|\sqrt{k}}}+\sqrt{\frac{k{\bar{\beta }}^{2}}{|w{|}^{2}}+1}$$

*and*

$$\bar{a}=\frac{\frac{k{\bar{\beta }}^{2}}{|w{|}^{2}}+1}{1+\frac{1}{\left|w\right|\sqrt{k}}}.$$

*Let $\delta =\frac{\epsilon }{b+\sqrt{b^{2}+2a\epsilon }}$ and $\bar{\delta }=\frac{\epsilon }{\bar{b}+\sqrt{{\bar{b}}^{2}+2\bar{a}\epsilon }}$*. *If* $\lambda \in E_{k}(v,\delta ,\alpha ,\beta )$ *and* $\bar{\lambda }\in E_{k}(w,\bar{\delta },\bar{\alpha },\bar{\beta })$ *then*

$$\left|\frac{\left(v+\lambda )\cdot (w+\bar{\lambda }\right)}{\left|v+\lambda \right|\left|w+\bar{\lambda }\right|}-\frac{v\cdot w}{\left|v\right|\left|w\right|}\right|\leq \epsilon .$$

**Proof**. Follows from Lemma A5 in Appendix A and the triangle inequality. □

We now need to understand the probability that any given entry of $\hat{v}$ (or $\hat{w}$) is close to the corresponding entry of *v* (or *w*). Since the number of walks arriving at a given node is binomial, we apply a Chernoff bound (Lemma 2) to the binomial distribution to obtain Corollary 1.

**Lemma 2** (Chernoff Bound [40]). *Let X ~ Binom*(*n*, *p*). *Let μ=E(X)=np*. *For δ* > 0,

$$Pr(X\leq (1-\delta )\mu )\leq exp\left(-\frac{\delta^{2}\mu }{2}\right)$$

*and*

$$Pr(X\geq (1+\delta )\mu )\leq exp\left(-\frac{\delta^{2}\mu }{2+\delta }\right).$$

**Corollary 1**. *Let X* ~ *Binom*(*n*, *p*). *For δ* > 0,

$$Pr\left(\left|\frac{X}{n}-p\right|>\delta p\right)\leq 2\cdot exp\left(-\frac{n\delta^{2}p}{2+\delta }\right)$$

*and*

$$Pr\left(\left|\frac{X}{n}-p\right|>\delta \right)\leq 2\cdot exp\left(-\frac{n\delta^{2}}{2p+\delta }\right).$$

Having bounded the probability of any one vector entry having small error, we now use a union bound to bound the probability that all entries have small error.

**Lemma 3**. *Fix n*, k∈ℕ. *Fix δ* ≥ 0 *and* 0 ≤ *α*, *β* ≤ 1. *Let v* = (*v*_1_, ···, *v*_*k*_) *such that v*_*i*_ ≥ 0 *and* ∑_*i*_ *v*_*i*_ = 1. *Let X*_*i*_ ~ *Binom*(*n*, *v*_*i*_) *such that* ∑_*i*_ *X*_*i*_ = *n*. *Let $\lambda_{i}=\frac{X_{i}}{n}-v_{i}$ and let λ* = (*λ*1, ···, *λ*_*k*_). *We have that*

$$Pr\left(\lambda \notin E_{k}(v,\delta ,\alpha ,\beta )\right)\leq 2k\;exp\left(-n\delta^{2}\cdot \text{min}\left\{\frac{\beta^{2}}{2\alpha +\delta \beta },\frac{\alpha }{2+\delta }\right\}\right)$$

**Proof**. Since *X*_*i*_ ≥ 0, *v*_*i*_ + *λ*_*i*_ ≥ 0. We now apply the Chernoff bound. For *v*_*i*_ ≥ *α*, we see that

$$Pr\left(\left|\lambda_{i}\right|\geq \delta v_{i}\right)=Pr\left(\left|\frac{X_{i}}{n}-v_{i}\right|\geq \delta v_{i}\right)\leq 2\cdot \text{exp}\left(-\frac{n\delta^{2}v_{i}}{2+\delta }\right)\leq 2\cdot \text{exp}\left(-\frac{n\delta^{2}\alpha }{2+\delta }\right)$$

For *v*_*i*_ <*α*, we see that

$$Pr\left(\left|\lambda_{i}\right|\geq \beta \delta \right)=Pr\left(\left|\frac{X_{i}}{n}-v_{i}\right|\geq \beta \delta \right)\leq 2\cdot \text{exp}\left(-\frac{n\beta^{2}\delta^{2}}{2v_{i}+\beta \delta }\right)\leq 2\cdot \text{exp}\left(-\frac{n\beta^{2}\delta^{2}}{2\alpha +\beta \delta }\right).$$

The result then follows by the union bound. □

Finally, we can combine the previous results to bound the required number of random walks, given error tolerance *∈* and success probability *r*.

**Lemma 4**. *Let ϵ* > 0 *and* 0 < *r* < 1. *For $c(\epsilon )=2{\left(c+\sqrt{c^{2}+2\epsilon }\right)}^{2}+\epsilon \left(c+\sqrt{c^{2}+2\epsilon }\right))$ and $C=\frac{5+4\sqrt{2}}{4}$*. *Let δ as in* Theorem 2. *After making n (non-dead-end) walks in the randomized pruned HeteSim algorithm*,

$$Pr\left(\lambda \notin E_{k}\left(v,\delta ,\frac{\left|v\right|}{\sqrt{k}},\frac{\left|v\right|}{\sqrt{k}}\right)\right)\leq 2k\;exp\left(-\frac{n}{k}\cdot \frac{\epsilon^{2}}{c(\epsilon )}\right).$$

**Proof**. We apply Lemma 3 and Theorem 2. We set $\alpha =\beta =\frac{\left|v\right|}{\sqrt{k}}\leq 1$ and $\delta =\frac{\epsilon }{b+\sqrt{b^{2}+2a\epsilon }}$. Thus,

$$Pr\left(\lambda \notin E_{k}(v,\delta ,\alpha ,\beta )\right)\leq 2k\text{ exp}\left(-n\cdot \frac{\left|v\right|}{\sqrt{k}}\cdot \frac{\epsilon^{2}}{2{\left(b+\sqrt{b^{2}+2a\epsilon }\right)}^{2}+\epsilon \left(b+\sqrt{b^{2}+2a\epsilon }\right)}\right).$$

We notice that the content of the exponent is a decreasing function in |*v*| (for |*v*| > 0). Thus,

$$Pr\left(\lambda \notin E_{k}(v,\delta ,\alpha ,\beta )\right)\leq 2k\text{ exp}\left(-\frac{n}{k}\cdot \frac{\epsilon^{2}}{2{\left(c+\sqrt{c^{2}+2\epsilon }\right)}^{2}+\epsilon \left(c+\sqrt{c^{2}+2\epsilon }\right)}\right)\text{. }$$

□

**Corollary 2**. *Under the same assumptions as* Lemma 4, *let* $n>\frac{c(\epsilon )}{\epsilon^{2}}\cdot k\text{ ln}\left(\frac{4k}{1-r}\right)$, *after making n (non-dead-end) walks in the randomized pruned HeteSim algorithm (on both sides of the computation)*,

$$Pr(\left|PHS(a,b|P)-\tilde{PHS}(a,b|P)\right|\epsilon )r.$$

**Proof**. Follows from Lemma 4 (applied to both sides of the computation), Theorem 2 and the union bound. □

**Remark 1**. *The exact number of walks required may differ due to the existence of walks that lead to dead end not counting*. *In* Appendix B, *we have provided some analysis of the probabilistic effects of this*.

#### 3.2.5. Deterministic Aggregation

In order to rank the overall relatedness of source nodes to a fixed target node, SemNet version 2 uses the mean HeteSim score between the source and target node, averaged over all metapaths which exist for any source node in the set under study.

For completeness, pseudocode for computing exact mean HeteSim scores is given in Algorithm 6.


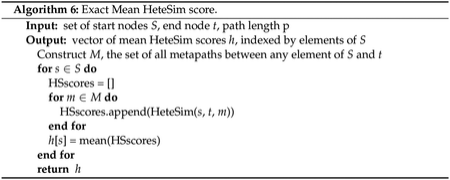


#### 3.2.6. Randomized Aggregation

As an alternative to taking the exact mean HeteSim score over all metapaths, we also consider an approximation to the mean given by the mean over a random subset of metapaths. Let *S* be a set of source nodes in the graph and *T* be a set of target nodes. Let $MP_{ST}$ be the set of all metapaths in the knowledge graph with at least one instance between some node in *S* and some node in *T*.Let (*s*, *t*) ∈ *S* × *T*. Let $P=A_{1}\overset{R_{1}}{\to }A_{2}\overset{R_{2}}{\to }\ldots \overset{R_{l}}{\to }A_{+1}$ by a metapath. Recall that HS(s,t∣P) is the HeteSim score between *s* and *t* relative to the metapath P. Similarly, let PHS(s,t∣P) be the Pruned HeteSim score between *s* and *t* relative to the metapath P.

The aggregated HeteSim score of a source–target pair(*s*, *t*) is defined to be

$$Q(s,t)=\frac{1}{\left|MP_{ST}\right|}\underset{P\in MP}{\sum }\text{HS}(s,t|P)$$

and the aggregated Pruned HeteSim Score is defined to be

$$R(s,t)=\frac{1}{\left|MP\right|}\underset{P\in MP}{\sum }\text{PHS}(s,t|P).$$

Notice that if we select a metapath from MP uniformly at random and took the HeteSim score relative to that metapath, the expected value of the score is precisely *Q*(*s*, *t*). Thus, we may approximate *Q*(*s*, *t*) by taking *m* independent and uniformly chosen math-paths, $P_{1},\cdots ,P_{m}$, and taking the mean of the HeteSim scores relative to these metapaths. Let

$$\hat{Q}(s,t)=\frac{1}{m}\overset{m}{\underset{i=1}{\sum }}HS\left(s,t|P_{i}\right).$$

Hence, $E(\tilde{R}(s,t))=R(s,t)$.

Let $\tilde{\text{PHS}}(s,t|P)$ be the approximation of PHS(s,t∣P) derived from our randomized algorithm after taking n(s,t∣P) random walks. Let k(s,t∣P) be the number of reachable nodes of type *A*_*l*/2+1_ when considering source *s*, target *t* and metapath P. Let $k_{\text{max}}=\text{max}\left\{k\left(s,t|P_{1}\right),\cdots ,k\left(s,t|P_{m}\right)\right\}$, for $MP_{ST}=\left\{P_{1},\ldots ,P_{m}\right\}$. By the construction of the algorithm, $E(\tilde{\text{PHS}}(s,t|P))=\text{PHS}(s,t|P)$ for a fixed P. Let

$$\tilde{R}(s,t)=\frac{1}{m}\overset{m}{\underset{i=1}{\sum }}\text{PHS}\left(s,t|P_{i}\right)$$

and

$$\hat{R}(s,t)=\frac{1}{m}\overset{m}{\underset{i=1}{\sum }}\tilde{\text{PHS}}\left(s,t|P_{i}\right).$$

Similarly to the above, $E(\tilde{R}(s,t))=R(s,t)$. We now see that

$$E(\hat{R}(s,t))=\frac{1}{m}\overset{m}{\underset{i=1}{\sum }}E\left(E\left(\tilde{\text{PHS}}\left(s,t|P_{i}\right)|P_{i}\right)\right)=E\left(\frac{1}{m}\overset{m}{\underset{i=1}{\sum }}\text{PHS}\left(s,t|P_{i}\right)\right)=E(\tilde{R}(s,t))=R(s,t).$$

We now provide bounds on the number of random metapaths (m) we require to have $\hat{Q}(s,t)$ and $\hat{R}(s,t)$ be within some error of *Q*(*s*, *t*) and *R*(*s*, *t*), respectively, with at least some probability.

**Lemma 5** (Bounded differences inequality [41]). *Let Z*_1_, ···, *Z*_*k*_ *be independent random variables such that Z*_*i*_ ∈ Λ_*i*_. *Let $f:\Lambda_{1}\times \cdots \times \Lambda_{k}\to \mathbb{R}$*. *Assume there exist $c_{1},\cdots ,c_{k}\in \mathbb{R}$ such that, for all i*,

$$\left|f\left(a_{1},\cdots ,a_{i-1},a_{i},a_{i+1},\cdots a_{k}\right)-f\left(a_{1},\cdots ,a_{i-1},a_{i}^{\prime },a_{i+1},\cdots a_{k}\right)\right|\leq c_{i}$$

*for all a*_*j*_ ∈ Λ_*j*_ *and $a_{i}^{\prime }\in \Lambda_{i}$*. *Let X* = *f*(*Z*_1_, ···, *Z*_*k*_). *We have that*

$$Pr(\left|X-E(X)\right|\geq t)\leq 2\;exp\left(-\frac{2t^{2}}{\sum_{i=1}^{k}c_{i}^{2}}\right).$$

**Lemma 6**. *For all* (*s*, *t*) ∈ *S* × *T*,

$$Pr(\left|\hat{Q}(s,t)-Q(s,t)\right|\geq \epsilon )\leq 2e^{-2m\epsilon^{2}}$$

*and*

$$Pr(\left|\tilde{R}(s,t)-R(s,t)\right|\geq \epsilon )\leq 2e^{-2m\epsilon^{2}}$$

*for all* (*s*, *t*) ∈ *S* × *T*.

**Proof**. Fix (*s*, *t*) ∈ *S*× *T*. We utilize the bounded differences inequality. We take $P_{1},\cdots ,P_{m}$ to be our independent random variables. Let

$$\hat{Q}\left(P_{1},\cdots P_{k}\cdots ,P_{m}\right)(s,t)=\frac{1}{m}\overset{m}{\underset{i=1}{\sum }}\text{HS}\left(s,t|P_{i}\right).$$

Notice that for any *k* ∈ [*m*],

$$\left|\hat{Q}\left(P_{1},\cdots P_{k}\cdots ,P_{m}\right)(s,t)-\hat{Q}\left(P_{1},\cdots P_{k}^{\prime }\cdots ,P_{m}\right)(s,t)\right|=\left|\frac{\text{HS}\left(s,t|P_{k}\right)-\text{HS}\left(s,t|P_{k}^{\prime }\right)}{m}\right|\;\leq \frac{1}{m}.$$

Thus, $C_{i}=\frac{1}{m}$ is sufficient to apply the bounded differences inequality. Hence,

$$Pr(\left|\hat{Q}(s,t)-E(\hat{Q}(s,t))\right|\geq \epsilon )\leq 2\text{ exp}\left(-\frac{2\epsilon^{2}}{\sum_{i=1}^{m}c_{i}^{2}}\right)=2e^{-2m\epsilon^{2}}.$$

Similar argument holds for $\tilde{R}(s,t)$. □

**Corollary 3**. *For $m=\frac{1}{2\epsilon^{2}}\text{ln}\left(\frac{2\left|S\right|\left|T\right|}{r}\right)$, with probability at least* 1 − *r*,

$$\left|\hat{Q}(s,t)-Q(s,t)\right|<\epsilon$$

*for all* (*s*, *t*) ∈ *S* × *T*.

**Proof**. Applying Lemma 6, we see that

$$Pr\left(\underset{(s,t)\in S\times T}{\cup }\left|\hat{Q}(s,t)-Q(s,t)\right|\geq \epsilon \right)\;\leq \underset{(s,t)\in S\times T}{\sum }Pr(|\hat{Q}(s,t)-Q(s,t))|\geq \epsilon )\;\leq 2\left|S\right|\left|T\right|e^{-2m\epsilon^{2}}.$$

Thus, the probability that $\left|\tilde{R}(s,t)-R(s,t)\right|<\epsilon$ for all (*s*, *t*) ∈ *S* × *T* is at least $1-2\left|S\right|\left|T\right|e^{-2m\epsilon^{2}}$. To have this probability at least 1 − *r*, it is hence sufficient to have $2\left|S\right|\left|T\right|e^{-2m\epsilon^{2}}=r$, proving the result. □

**Theorem 3**. *Fix* 0 < *ϵ*, *r* < 1. *For*

$$n\left(s,t|P_{i}\right)=\frac{4c\left(\frac{\epsilon }{2}\right)\cdot k\left(s,t|P_{i}\right)}{\epsilon^{2}}\text{ln}\left(\frac{4m\left|S\right|\left|T\right|k_{\text{max}}}{r_{1}}\right)$$

*and*

$$m=\frac{2}{\epsilon^{2}}\text{ln}\left(\frac{2\left|S\right|\left|T\right|}{r-r_{1}}\right),$$

*where $r_{1}=r\cdot \frac{4\text{ ln}\left(\frac{2\left|S\right|\left|T\right|}{r}\right)k_{\text{max}}}{4\text{ ln}\left(\frac{2\left|S\right|\left|T\right|}{r}\right)k_{\text{max}}+\epsilon^{2}}$, with probability at least* 1 − *r*,

$$\left|\hat{R}(s,t)-R(s,t)\right|<\epsilon$$

*for all* (*s*, *t*) ∈ *S* × *T*.

The proof of this result is deferred to Appendix A.

The results from this section give rise to 2 algorithms for computing approximations to mean HeteSim scores. First, Corollary 3 gives an algorithm for approximating the mean HeteSim score using the deterministic HeteSim algorithm given in Algorithm 1. Pseudocode for this approximate mean HeteSim computation is given in Algorithm 7. Second, Theorem 3 shows how to compute an approximation to the mean pruned HeteSim score, and pseudocode for this computation is given in Algorithm 8.


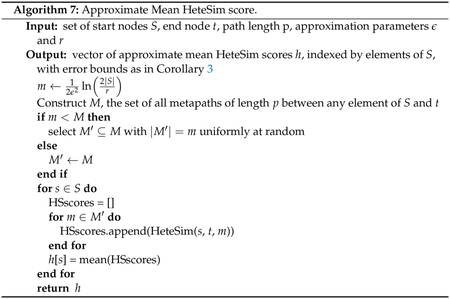


### 3.3. Algorithm Runtimes: SemNet Version 2

Having given algorithms and proofs of correctness, we now turn to a computational investigation of actual algorithm performance. Our emphasis is on comparing the three different algorithms enumerated above.

#### 3.3.1. Verification of Randomized Algorithm Performance

**Figure 8.**
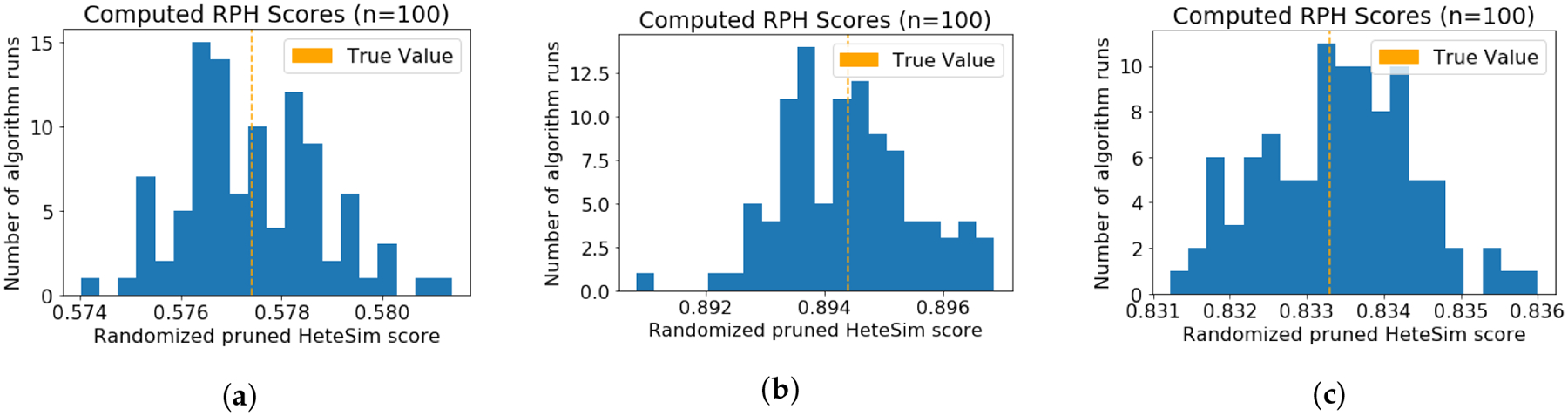
Computed randomized pruned HeteSim (RPH) scores for each of the three test graphs. (**a**) Test graph 1; (**b**) Test graph 2; (**c**) Test graph 3.

For each of the three test graphs and corresponding metapaths, the randomized pruned HeteSim algorithm was run 100 times, with *ϵ* = 0.05 and *r* = 0.95. For each of the three test graphs, an error less than *ϵ* was observed in all 100 iterations. Histograms showing the distribution of computed values are given in Figure 8.


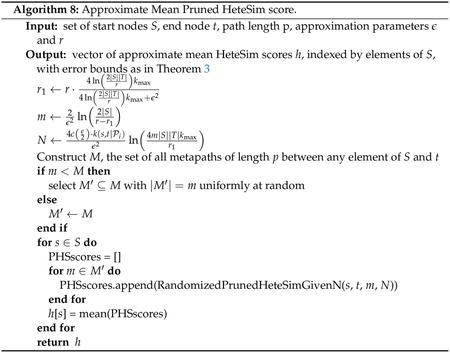


#### 3.3.2. Comparison of Algorithm Runtimes

For two of the three main algorithm variants, runtime on length 2 metapaths was measured, using Alzheimer’s disease as a target node and a set of three source nodes: insulin, hypothyroidism, and amyloid. Each of these source nodes has some amount of real-world domain significance; all three have, at some point, acted as a source node to the target node Alzheimer’s disease in other ongoing research in the authors’ lab. This ongoing work aims to investigate and discover causes and treatments (re-purposed or otherwise) within the active body of biomedical academic literature. As a more specific example, SemNet version 1 was used to investigate how hypothyroidism and Alzheimer’s disease are related via the combined rankings of shared source nodes. This is a slightly different application than what is being investigated in this manuscript, but the results definitively show that hypothyroidism and Alzheimer’s disease are closely related. These previous runs have historically been extremely slow while utilizing SemNet version 1, taking up to an hour to complete (see Table 2). Decreasing runtime is the main motivation for the new algorithms and implementations.

For the two main chosen algorithms associated with SemNet version 2, mean exact HeteSim and approximate mean HeteSim, test runs were conducted using the previously defined source–target combinations. These test runs were repeated 10 times per combination for both algorithms respectively, and the comprehensive runtime results can be seen in Table 3. For approximate mean HeteSim, the realistic parameters *ϵ* = 0.1 and *r* = 0.9 were used. The third algorithm variant, approximate mean pruned HeteSim, was not run on the actual knowledge graph, due to excessive runtime when using realistic values for *ϵ* and *r*.

For the fastest algorithm, approximate mean HeteSim, time spent on each of the three steps described in Figure 5 was also recorded. To further accentuate the speed differences between SemNet versions 1 and 2 (specifically approximate mean HeteSim), Table 2 shows the three step breakdown for both SemNet versions side by side. For both versions, the same target (Alzheimer’s disease) and sources (insulin, hypothyroidism, and amyloid) were used, and each source–target combination, like in Table 3, was run 10 times each. The runtime ratio between SemNet version 1 and SemNet version 2 is also shown in Table 2. For these three step breakdown tests, the approximate mean HeteSim algorithm used the parameters *ϵ* = 0.1 and *r* = 0.9 once again.

**Figure 9.**
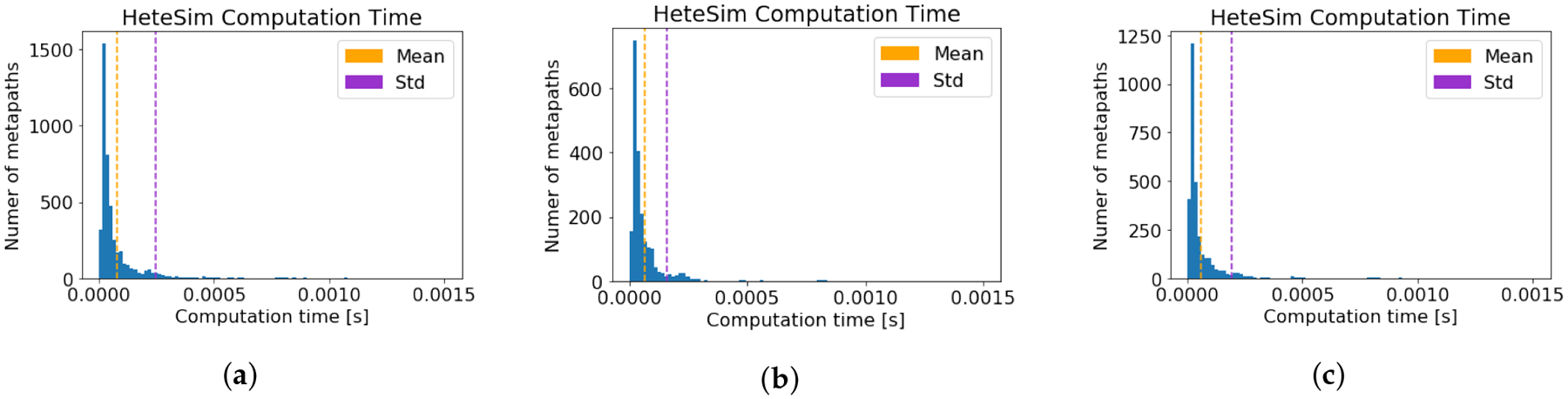
HeteSim computation times per metapath for all metapaths of length 2 from the given source node to Alzheimer’s disease, using the deterministic HeteSim implementation from SemNet version 2. (**a**) Insulin; (**b**) Hypothyroidism; (**c**) Amyloid.

Additionally, the time to compute HeteSim using the new data structure for a single metapath was analyzed. Due to the HeteSim algorithms being run on single metapaths, aggregation (Step 3) was not used and therefore not represented in timing results. For comparison, the top 20 unique metapaths, based on the metapaths with the highest number of unique paths (each metapath between a source and target node can potentially encompass many different paths), were used as inputs to the respective algorithms. Both the deterministic HeteSim and randomized pruned HeteSim algorithms were run on these metapaths, with approximation parameters *ϵ* = 0.1 and *r* = 0.9 applied to the latter. Randomized pruned HeteSim was not run on all metapaths due to excessive runtime, and, therefore, deterministic HeteSim was also not run over all metapaths, for comparison sake. Results of this comparison are given in Table 4. Further detail on the randomized pruned HeteSim results, including the maximum and minimum values for the number of iterations, runtimes, and metapath instances (the number of paths within a metapath), is given in Table 5. Figure 9 shows the breakdown of deterministic HeteSim computation time for each metapath between the described sources and target, with no limit on the number of metapaths.

As a final timing comparison, the top 20 length 4 metapaths (again determined by the metapaths comprised of the highest number of unique paths) were generated for each of the three testing target-source node pairs, and the deterministic HeteSim algorithm was run on all 60 metapaths. The 20 length 4 metapaths were taken out of a subset of the first 100,000 total length 4 metapaths shared between each respective source node and AD. Metapath computation is the greatest bottleneck, and retrieving any more than 100,000 metapaths per source node is simply too time consuming as of right now. The maximum, minimum, and mean runtimes for this final test are shown in Table 6. As a final side note, different runs of both SemNet versions 1 and 2 might vary in computational time due to changes in concurrent computational load and random, extrinsic factors. This slight variation does not change the ultimate goal or conclusion of this study.

### 3.4. Study Assessing User Friendliness of SemNet Version 2

SemNet version SemNet version 1 had extensive Sphinx documentation and readme files, but there was no detailed example Jupyter interface for users with limited computer science or Python background to easily run the software. User friendliness was primarily assessed with a standardized survey of two distinct groups of naive or first-time SemNet software users who were trained in either SemNet version 1 or SemNet version 2. “Training” included a general introduction or background on the purpose and utility of the SemNet framework (same content for each group), along with publicly available user documentation (documentation to either SemNet version 1 or SemNet version 2, depending on user group assignment). The SemNet version 1 user group had 11 users (n = 11), whereas the SemNet version 2 user group had 10 users (n = 10). All participants were students at Georgia Institute of Technology.

To ensure that differences in prior experience with Python or Jupyter notebooks would not bias the user study results, each participant was asked to self-classify their prior experience using Python and/or Jupyter notebooks to ensure each user group had a balanced distribution of prior Python/Jupyter user experiences. The Python experience classifications were: novice user (no to minimal Python experience); proficient user (had taken a basic Python class or had previously independently used Python for an elementary project); or expert Python user (very confident and capable of teaching a class on Python/Jupyter). The SemNet version 1 group included 3 participants who self-identified as novice Python users, 7 that self-identified as proficient Python users, and 1 that self-identified as an expert Python user. The SemNet version 2 group included 3 participants who self-identified as novice Python users, 6 that self-identified as proficient Python users, and 1 that self-identified as an expert Python user.

After completing a standardized training protocol, each user took an electronic survey asking a simple question: “Are you comfortable in running a [SemNet] simulation on your own?”. The SemNet version 1 group had 2 of 11 users who answered they were comfortable in running a SemNet version 1 simulation after minimal training. The SemNet version 2 group had 8 of 10 users that answered they were comfortable in running a SemNet version 2 simulation after minimal training. Fisher’s exact test compared these two user groups; the SemNet version 2 user group was significantly (*p* < 0.05) more comfortable performing a simulation compared to users in the SemNet version 1 group. This result quantitatively affirms that the SemNet version 2 framework is more user friendly and intuitive than SemNet version 1.

Finally, a random subset of users (n = 7) were eventually trained in both SemNet version 1 and SemNet version 2. These users were asked a simple question via an electronic survey: “Is the user friendliness of SemNet version 2 equal, somewhat better, or much better than SemNet version 1?” All 7 users said SemNet version 2 was “much better” than SemNet version 1. While the sample size is small, the probability that all 7 users select “much better” is significant (*p* < 0.05). SemNet version 2’s interface and greatly enhanced speed were the volunteered reasons stated for it being voted “much better” by users for its user friendliness.

### 3.5. Assessing Highly Ranked Metabolic Nodes to Alzheimer’s Disease

Recent literature has identified relationships shared between metabolic co-morbidities and AD [33,42,43]. The scope of the present article focuses on the mathematics, computational optimizations, performance improvements, and user friendliness of SemNet version 2. An entirely different manuscript could be dedicated to sifting through interesting results on the Alzheimer’s case study used to perform SemNet version 2 performance evaluations. Due to space constraints and article scope, we only briefly touch on some of the interesting nodes identified and ranked in SemNet version 2 using Alzheimer’s disease (AD) as the target node and hypothyroidism and insulin as source nodes of interest.

One of the key advantages of SemNet is examining multi-factorial relationships that are not as obvious. A small subset of lesser discussed source nodes involving metabolic co-morbidities and AD ranked as relatively important by SemNet version 2 include the following: metformin (a drug used to treat type 2 or adult-onset diabetes), dexamethasone (a glucocorticoid use to treat inflammation, autoimmune disease, or adrenal insufficiency), carbonic anhydrase (a family of enzymes that catalyze the interconversion between carbon dioxide and water), and nitric oxide synthase 3 (generates NO in blood vessels and is involved with regulating vascular function). These specific source nodes are identified by finding all intersecting source nodes shared between AD and multiple targets (metabolic co-morbidities, in this example) and ranking all shared sources with respect to each AD-metabolic co-morbidity pairing. In this example, the chosen metabolic co-morbidities associated with AD are obesity, hypothyroidism, and type 2 diabetes [34,35,44]. The four example source node results mentioned above (metformin, dexamethasone, carbonic anhyrdrase, and nitric oxicde 3) scored very highly in each run of SemNet version 2, consistently placing in the top 25% of ranked nodes based on HeteSim score. More specific explanations for why or how these identified source nodes are tied to AD are discussed in studies contributing to the knowledge graph connectivity, some of which are cited here [45–48].

## 4. Discussion

The results presented in this manuscript show that the main objective, reducing SemNet’s overall runtime, has been achieved. This increase in speed is attributable to both algorithmic improvements (best seen with the approximate mean HeteSim algorithm) and, most substantially, data structure changes. The secondary objective, fixing the error presented in the SemNet version 1 rank aggregation algorithm ULARA, was also met with the introduction of two new aggregation algorithms: exact mean aggregation and approximate mean aggregation. The success presented in this work will provoke a quick adoption of SemNet version 2. Computational challenges still remain, specifically in metapath enumeration and computation. The need to compute all metapaths between the specified source–target nodes is still a relatively major computational bottleneck to be addressed in future work.

### 4.1. Computational Improvements

Both the mean HeteSim score and approximate mean HeteSim score show runtime reductions compared to SemNet version 1. These improvements are evident both in the overall algorithm runtimes (Tables 1 and 3) and in the speed of the deterministic HeteSim subroutine (Tables 1 and 4). Note that, though the number of metapaths decreased in the graph used to test SemNet version 2 and this reduction must account for some speedup, computation time per metapath decreased. Table 2 shows that the largest improvement happened in step 2, likely because the implementation of step 2 in SemNet version 1 used many Neo4j queries. Since it has already been shown that Neo4j queries made up most of the runtime in SemNet version 1 (see Table 1), it is likely that the substitution of the Python dictionary-based data structure for the knowledge graph was the largest source of runtime reduction for step 2. Similarly, step 1 involves querying the knowledge graph, and the replacement of Neo4j with a custom dictionary-based data structure is likely the largest source of improvement here as well.

Step 3 is a bit different because the changes here were motivated by the replacement of a flawed rank aggregation technique, rather than runtime considerations. As a ratio, we do see an improved reduction in runtime of over 1000, but the absolute runtime values for step 3 are quite small in relation to the entire algorithm. The most important result regarding step 3 is the replacement ULARA with a sensible alternative (mean HeteSim score) that is also is amenable to approximation based on randomization. In the length 2 metapath tests reported in Table 3, the approximate mean HeteSim algorithm achieves a 20% runtime reduction compared to the exact mean HeteSim score computation. This reduction is mostly attributable to the need to run the HeteSim subroutine on fewer metapaths. Since the bound on the number of metapaths for which HeteSim must be computed depends only on the number of candidate source nodes and the approximation parameters *ϵ* and *r* (see Corollary 3), the performance advantage of the approximate mean computation should be even more substantial in situations involving more metapaths. This performance advantage will only become more pronounced when running the approximate mean HeteSim algorithm on longer metapaths because, generally, the longer the metapath the greater the instances of that metapath within the graph. As a final note, the use of approximation algorithms, or more tangibly the tradeoff of some accuracy for a large performance boost, is appropriate in this context. This conclusion is drawn from two generalizations: the knowledge graph is inherently noisy, as it is generated using natural language processing techniques on biomedical paper abstracts, and the primary use of SemNet is in hypothesis generation. Both factors make the accuracy/speed tradeoff an allowable, and generally preferable, possibility that might not be available in different contexts.

### 4.2. Mathematical Limitations

In Corollary 2, we provide a bound that demonstrated that it is sufficient to make $O\left(\frac{1}{\epsilon^{2}}k\text{ ln}\left(\frac{k}{1-r}\right)\right)$ random walks in the randomized Pruned HeteSim algorithm. As illustrated by Table 5, the bound we achieved may, at times, result in a large number of required walks, when considering realistic knowledge graphs and modest values for *ϵ* and *r*. We acknowledged that the bound we achieved may be crude, especially in our frequent use of the, generally loose, union bound. Hence, we leave open the possibility of substantial improvement to both the constant we achieve (*c*(*ϵ*) ≤ 71) and the order with respect to the various variables.

One possible area of improvement is in the order with respect to *k*. We conjecture that the required number of walks is at least order *k*, thus leaving room for the possibility of the true value to be between order *k* and *k* log *k* (inclusive). Considering the order with respect to *ϵ*, we note that most standard general concentration inequalities necessitate $O\left(\frac{1}{\epsilon^{2}}\right)$. This being said, the distribution we are considering is binomial. While the authors are not aware of any stronger results for the binomial distribution, we are also not aware of any reason why such a result could not exist.

We also note that to achieve Lemma 4, we utilize an error allocation scheme that bounds large entries with error proportional to the value of the entry but bounds small entries with a fixed bound. This is just one possible scheme which leaves open the possibility of achieving tighter results using another, possibly more individualized, scheme.

### 4.3. Limitations and Future Directions

The knowledge graph used to test SemNet version 2 has substantially fewer edges than the knowledge graph used in SemNet version 1, as seen by the reduced number of metapaths between vertices of interest (see Tables 1 and 2). The new graph was built to reduce the number of overly generic edges and redundant conclusions occasionally seen in SemNet version 1; the new graph is, overall, both better performing and more useful for hypothesis generation compared to the old graph. Future work will address this limitation and give more accurate runtime comparisons by building a knowledge graph of comparable size to that used in SemNet version 1, though this endeavor would mostly just be a confirmatory effort to give more precise runtime improvements.

Though the new implementation has significantly reduced the runtime required to enumerate metapaths, metapath enumeration remains a computational bottleneck. This bottleneck is a barrier to HeteSim computations on longer metapaths; this work has made length 4 metapath analysis feasible, though anything greater is potentially still unattainable. Since counting the number of paths between two specified nodes in a directed graph is #**P**-complete [49], metapath enumeration is likely also a computationally hard problem. To make further progress, future work will need to address this metapath enumeration problem. One possible approach is to devise an algorithm for sampling metapaths under a uniform (or other useful) probability distribution, perhaps using a Markov chain Monte Carlo technique similar to the approach employed in [50]. If such an algorithm could be devised, it could be used directly with the randomized aggregation scheme described in Algorithm 7.

### 4.4. Related Work

In this section, SemNet version 2 (i.e., SemNet 2.0) is compared and contrasted to other existing automated LBD tools.

#### 4.4.1. Biomedical Knowledge Graphs

While a number of companies boast commercial biomedical knowledge bases, most publicly available KBs are limited in scope and diversity of node types. Many of these are created by aggregating specific, high-quality databases together. Databases in this category include Hetio [51], a KG built for drug re-purposing containing 48 K, 2.2 M edges, and 22 node types; OGB-BioKG from Open Graph Benchmark [52], a general-purpose biomedical KG containing 93 K nodes, 5 M edges, 5 node types, and 51 edge types (most of which are specific drug-drug interactions); DRKG [53] is a drug repositioning knowledge graph for COVID-19 that combines entities/relations from 6 existing databases with additional entity and relationship data extracted from open-source biomedical literature on COVID-19.

Other biomedical KGs have been created by using natural language processing to extract information from biomedical text. PubMed Knowledge Graph [54], which creates a paper-centric knowledge graph by linking authors, entities, institutions, and funding sources to research articles and connecting articles via citations. SemMedDB [8] contains a approximately 100 M (subject, object, predicate) triples extracted from PubMed articles from 124 node types and 58 relation types, each of which is linked to the article from which it was taken. SemNet 2.0’s knowledge graph is derived from a processed version of SemMedDB which removes links to papers and aggregates relation triples to more directly identify the relationships between biological entities.

#### 4.4.2. Related Algorithms

At its core, SemNet 2.0 is a framework for identifying relatedness among nodes in a knowledge graph. This is similar to other knowledge base completion (KBC) algorithms, which seek to identify missing edges between knowledge graph nodes. A large family of knowledge base completion algorithms seek to infer missing edges by modeling entity and relation representations as latent embeddings and learning these by encourage them to satisfy certain geometric properties. For example, TransE treats each entity as a point in Euclidean space and assumes that relations can be effectively modeled as translations between entity embeddings, i.e., *s* + *r* ≈ *t* for source node *s*, target node *t*, and relation *r*. A wide variety of other models operate on some variant of this assumption, substituting translation by element-wise scaling [55], rotation in complex space [56], rotation in Quaternary space [57], or rotation and reflection in hyperbolic space [58]. An smaller, alternative family of knowledge base completion literature focuses instead on inferring missing relations by aggregating information either explicitly [59] or implicitly [60,61] encoded in the (meta)paths between them. This approach is more desirable for biomedical KBs due to the fact that relevant nodes and paths can be extracted from the graph to provide an understandable explanation of the predictions. SemNet 2.0 is most similar to this family of path-based KBC models but differs in that SemNet 2.0 computes a general measure of relatedness instead of predicting the specific type of relation between KB entities.

## 5. Conclusions

In conclusion, with novel biomedical research constantly being generated and computational power ever increasing, literature-based discovery is here to stay. LBD is a field that will only become more relevant as time goes on, but for it to achieve user adoption at a large scale, tools and methods must be created that allow for efficient LBD to take place. SemNet, a tool that was first developed in 2019, is a novel attempt at performing LBD with an approach that, up to this point, has rarely been observed. SemNet departs from existing attempts by being both domain agnostic and simple to use, two features uncommon in current LBD systems. These features enable users of SemNet to quickly navigate the comprehensive biomedical concept graph and begin generating ranked lists of concepts that will ultimately facilitate new hypothesis generation. SemNet version 1 was the first iteration of SemNet, and it largely succeeded at being both an LBD tool and a general-purpose starting point for essentially any biomedical investigation that relies, in some capacity, on literature-based data. Through widespread, practical adoption, potential improvements for SemNet version 1 became apparent, particularly regarding runtime and HeteSim score aggregation for source nodes. SemNet version 2 (i.e., SemNet 2.0) addresses these problems in three predominant ways: an improved graph data structure, improved HeteSim implementations, and improved HeteSim score aggregation. With these advancements, SemNet 2.0 is a major step forward in improving the efficiency and efficacy of interactive automated LBD tools.

SemNet 2.0 has been compiled into a Python package. This package, along with the SemMedDB data required to build the biomedical concept graph, is open source. Detailed documentation has been included with the package, all of which can be downloaded on GitHub.

## Figures and Tables

**Table 1.** SemNet version 1 HeteSim computation times for all metapaths between each of the three source nodes and Alzheimer’s disease.

Source Node	Insulin	Hypothyroidism	Amyloid
Number of metapaths	4873	2148	3095
Total computation time (min)	93.7	39.7	55.4
Computation time per metapath (s) (±std)	46.0 ± 6.1	44.2 ± 3.8	42.8 ± 4.2
Neo4j query time, per metapath (s) (±std)	44.9 ± 4.6	43.2 ± 2.8	42.1 ± 3.5
Time per metapath, excluding query time (s) (±std)	1.1 ± 3.1	1.0 ± 2.3	0.8 ± 1.9

**Table 2.** Mean and standard deviation of runtimes for both SemNet version 1 and the approximate mean HeteSim algorithm from SemNet version 2, broken down by step as in Figure 5.

Source Node	Insulin	Hypothyroidism	Amyloid
Num metapaths (SemNet 1)	4873	2148	3095
SemNet 1: Step 1 (s)	81 ± 5.3	35 ± 2.4	84 ± 5.3
SemNet 1: Step 2 (s)	220,000 ± 2300	96,000 ± 270	220,000 ± 2700
SemNet 1: Step 3 (s)	0.80 ± 0.0021	0.39 ± 0.0093	0.80 ± 0.014
Num metapaths (SemNet 2)	4521	2130	3060
SemNet 2: Step 1 (s)	1.2 ± 0.0093	0.19 ± 0.0015	0.41 ± 0.0024
SemNet 2: Step 2 (s)	0.0047 ± 0.00097	0.0026 ± 0.00060	0.0027 ± 0.00061
SemNet 2: Step 3 (s)	8.3 × 10^−5^ ± 1.4 × 10^−6^	8.3 × 10^−5^ ± 1.9 × 10^−6^	8.3 × 10^−5^ ± 1.2 × 10^−6^
Runtime ratio: Step 1	68	184	200
Runtime ratio: Step 2	4.7 × 10^8^	3.6 × 10^7^	8.1 × 10^7^
Runtime ratio: Step 3	9600	470	9600

**Table 3.** Mean and standard deviation of runtimes for the mean exact HeteSim and approximate mean HeteSim algorithms.

Algorithm	Runtime (s)
Mean exact HeteSim	4.1 ± 0.060
Approximate mean HeteSim	3.9 ± 0.015

**Table 4.** Mean and standard deviation of runtimes for both the deterministic HeteSim and randomized pruned HeteSim algorithms on the top 20 individual length 2 metapaths.

Source Node	Deterministic HeteSim	Randomized Pruned HeteSim
Insulin	2.0 × 10^−3^ ± 1.2 × 10^−3^	3500 ± 3400
Hypothyroidism	7.2 × 10^−4^ ± 3.4 × 10^−4^	440 ± 650
Amyloid	9.9 × 10^−4^ ± 6.4 × 10^−4^	1200 ± 1200

**Table 5.** Computation details for the randomized pruned HeteSim algorithm on the top 20 individual length 2 metapaths.

Source Node	Insulin	Hypothyroidism	Amyloid
Max iterations (*N*)	28,019,926	8,547,987	12,790,378
Min iterations (*N*)	5,308,942	1,666,564	3,229,242
Mean iterations (*N*)	10,068,473	2,632,969	5,206,723
Max runtime (s)	14,588	3138	5052
Min runtime (s)	420	99	247
Mean runtime (s)	3491	438	1193
Max metapath instances	488	167	240
Min metapath instances	109	39	70

**Table 6.** Maximum, minimum, and mean runtimes (with standard deviation) for the SemNet version 2 deterministic HeteSim algorithm on the top 20 individual length 4 metapaths.

Source Node	Insulin	Hypothyroidism	Amyloid
Max runtime (s)	0.21	0.022	0.033
Min runtime (s)	0.032	0.0029	0.0070
Mean runtime (s) (±std)	0.11 ± 0.039	0.011 ± 0.0056	0.015 ± 0.0075

## Data Availability

SemNet 2.0 code can be found on GitHub https://github.com/pathology-dynamics/semnet-2 (accessed on 10 January 2022). Access instructions to SemRep/SemMedDB/SKR Resources for non-commercial use can be found at https://lhncbc.nlm.nih.gov/ii/tools/SemRep_SemMedDB_SKR.html (accessed on 10 January 2022).

## Appendix A

### Appendix A.1. Technical Lemmas

**Lemma A1**. *For $v\in S_{k}$, $\frac{1}{\sqrt{k}}\leq \left|v\right|\leq 1$*.

**Proof**. By method of Lagrange Multipliers. □

**Lemma A2**. *Let δ* > 0*, α* > 0 *and* 0 < *β* ≤ 1. *Let v*, $w\in S_{k}$ *and $\lambda \in E_{k}(v,\delta ,\alpha ,\beta )$*. *We have that*

$$\left|\lambda \cdot v\right|\leq \delta \left(|v{|}^{2}+\frac{k\beta^{2}}{4}\right)\;and\;\left|\lambda \cdot \frac{w}{\left|w\right|}\right|\leq \left|\lambda \right|\leq \delta \sqrt{k\beta^{2}+|v{|}^{2}}.$$

**Proof**. Assume *v*_*i*_ has *m* entries less than *α* and these are the first *m* entries. Clearly, *m* ≤ *k*. We see that

$$\lambda \cdot v\leq \overset{k}{\underset{i=1}{\sum }}\lambda_{i}\cdot v_{i}\;\leq \overset{m}{\underset{i=1}{\sum }}\beta \delta \cdot v_{i}+\overset{k}{\underset{i=m+1}{\sum }}\delta \cdot v_{i}^{2}\;\leq \delta \overset{m}{\underset{i=1}{\sum }}v_{i}\left(\beta -v_{i}\right)+\delta \overset{k}{\underset{i=1}{\sum }}\cdot v_{i}^{2}\;\leq \delta |v{|}^{2}+\delta \frac{k\beta^{2}}{4}.$$

We obtain the lower bound similarly. Clearly, *m* ≤ *k*. Thus, we also see that

$$\left|\lambda \cdot \frac{w}{\left|w\right|}\right|\leq |\left|\lambda |\cdot \frac{\lambda }{\left|\lambda \right|}\cdot \frac{w}{\left|w\right|}\right|\leq \left|\lambda \right|\;\leq \sqrt{\overset{m}{\underset{i=1}{\sum }}{\left(\beta \delta \right)}^{2}+\delta^{2}\overset{k}{\underset{i=m+1}{\sum }}v_{i}^{2}}\;\leq \delta \sqrt{k\beta^{2}+|v{|}^{2}}.$$

□

**Lemma A3**. *For v*, $\lambda \in {\mathbb{R}}^{k}$ *such that v*, $v+\lambda \in S_{k}$,

$$\left|\left|v+\lambda \right|-\left|v\right|\right|\leq \frac{2\left|\lambda \cdot v\right|+|\lambda {|}^{2}}{\left|v\right|+\frac{1}{\sqrt{k}}}.$$

**Proof**. We first see that

$$∥v+\lambda |-|v||=\frac{∥v+\lambda |+|v||\cdot ∥v+\lambda |-|v||}{∥v+\lambda |+|v||}\;=\frac{||v+{\lambda |}^{2}-|v{|}^{2}|}{∥v+\lambda |+|v||}\;\leq \frac{||v+{\lambda |}^{2}-|v{|}^{2}|}{|v|+\frac{1}{\sqrt{k}}}.$$

We now see that

$$\left|v+\lambda {|}^{2}=(v+\lambda )\cdot (v+\lambda )=v\cdot v+2\lambda \cdot v+\lambda \cdot \lambda \;=\right|v{|}^{2}+2\lambda \cdot v+|\lambda {|}^{2}$$

$$\left|v+\lambda {|}^{2}-\right|v{|}^{2}\leq 2\left|\lambda \cdot v\right|+|\lambda {|}^{2}.$$

Similarly, we get that |*v*|^2^ − |*v* + *λ*|^2^ ≤ 2|*λ* · *v*| + |*λ*|^2^. The desired result then follows. □

**Lemma A4**. *Let β* ≤ 1. *Let* 0 < *δ and α*, *β* ≥ 0. *For* $v\in S_{k}$ *and* $\lambda \in E_{k}(v,\delta ,\alpha ,\beta )$,

$$\left|\frac{(v+\lambda )\cdot w}{\left|v+\lambda \right|\left|w\right|}-\frac{v\cdot w}{\left|v\right|\left|w\right|}\right|\leq \delta \left(2+\frac{k\beta^{2}}{2|v{|}^{2}}+\sqrt{\frac{k}{|v{|}^{2}}\beta^{2}+1}\right)+\delta^{2}\left(\frac{k\beta^{2}}{|v{|}^{2}}+1\right).$$

**Proof**. We see that

$$\frac{(v+\lambda )\cdot w}{\left|v+\lambda \right|\left|w\right|}-\frac{v\cdot w}{\left|v\right|\left|w\right|}=\frac{(v+\lambda )\cdot w}{\left|v+\lambda \right|\left|w\right|}-\frac{(v+\lambda )\cdot w}{\left|v\right|\left|w\right|}-\frac{\lambda \cdot w}{\left|v\right|\left|w\right|}\;=\frac{(v+\lambda )\cdot w}{\left|w\right|}\left(\frac{1}{\left|v+\lambda \right|}-\frac{1}{\left|v\right|}\right)-\frac{\lambda \cdot w}{\left|v\right|\left|w\right|}\;=\frac{(v+\lambda )\cdot w}{\left|v+\lambda \right|\left|w\right|}\cdot \frac{\left|v\right|-\left|v+\lambda \right|}{\left|v\right|}-\frac{1}{\left|v\right|}\cdot \left(\lambda \cdot \frac{w}{\left|w\right|}\right)$$

$$\left|\frac{(v+\lambda )\cdot w}{\left|v+\lambda \right|\left|w\right|}-\frac{v\cdot w}{\left|v\right|\left|w\right|}\right|\leq \frac{1}{\left|v\right|}\left(\left|\left|v\right|-\right|v+\lambda ||+\left|\lambda \cdot \frac{w}{\left|w\right|}\right|\right)\;\leq \frac{2\delta \left(|v{|}^{2}+\frac{k\beta^{2}}{4}\right)+\delta^{2}\left(k\beta^{2}+|v{|}^{2}\right)}{|v{|}^{2}+\frac{\left|v\right|}{\sqrt{k}}}+\delta \sqrt{\frac{k\beta^{2}}{|v{|}^{2}}+1}\;\leq \frac{\delta \left(2+\frac{k\beta^{2}}{2|v{|}^{2}}\right)+\delta^{2}\left(\frac{k\beta^{2}}{|v{|}^{2}}+1\right)}{1+\frac{1}{\left|v\right|\sqrt{k}}}+\delta \sqrt{\frac{k\beta^{2}}{|v{|}^{2}}+1}.$$

The above inequality follows from $\left|\frac{v\cdot w}{\left|w\right|\left|v\right|}\right|\leq 1$, Lemmas A2 and A3. □

**Lemma A5**. *Fix ϵ*. *Let β* ≤ 1 *and α* ≥ 0. *For $v\in S_{k}$ and $\lambda \in E_{k}\left(v,\alpha ,\delta ,\delta^{\prime }\right)$*. *Let*

$$b=\frac{2+\frac{k\beta^{2}}{2|v{|}^{2}}}{1+\frac{1}{\left|v\right|\sqrt{k}}}+\sqrt{\frac{k\beta^{2}}{|v{|}^{2}}+1}\;and\;a=\frac{\frac{k\beta^{2}}{|v{|}^{2}}+1}{1+\frac{1}{\left|v\right|\sqrt{k}}}.$$

*For* $\delta \leq \frac{\epsilon }{b+\sqrt{b^{2}+2a\epsilon }}$,

$$\left|\frac{(v+\lambda )\cdot w}{\left|v+\lambda \right|\left|w\right|}-\frac{v\cdot w}{\left|v\right|\left|w\right|}\right|\leq \frac{\epsilon }{2}.$$

**Proof**. The result follows from Lemma A4. □

### Appendix A.2. Proofs and Theorems

**Proof**. For *ϵ*_1_, *ϵ*_2_ > 0 such that *ϵ*_1_ + *ϵ*_2_ = *ϵ*, we see that

$$Pr(\left|\hat{R}(s,t)-R(s,t)\left|\geq \epsilon )\leq Pr(\right|\hat{R}(s,t)-\tilde{R}(s,t)\left|+\right|\tilde{R}(s,t)-R(s,t)\right|\geq \epsilon )$$

$$Pr(\left|\hat{R}(s,t)-R(s,t)\left|\leq \epsilon )\geq Pr(\right|\hat{R}(s,t)-\tilde{R}(s,t)\left|+\right|\tilde{R}(s,t)-R(s,t)\right|\leq \epsilon )\;\geq Pr\left(\left|\hat{R}(s,t)-\tilde{R}(s,t)\left|\leq \epsilon_{1}\cap \right|\tilde{R}(s,t)-R(s,t)\right|\leq \epsilon_{2}\right)$$

$$Pr(\left|\hat{R}(s,t)-R(s,t)\right|\geq \epsilon )\leq Pr\left(\left|\hat{R}(s,t)-\tilde{R}(s,t)\left|\geq \epsilon_{1}\cup \right|\tilde{R}(s,t)-R(s,t)\right|\geq \epsilon_{2}\right)\;\leq Pr\left(\left|\hat{R}(s,t)-\tilde{R}(s,t)\right|\geq \epsilon_{1}\right)+Pr\left(\left|\tilde{R}(s,t)-R(s,t)\right|\geq \epsilon_{2}\right).$$

Recall from Lemma 6 that

$$Pr\left(\left|R(s,t)-\tilde{R}(s,t)\right|\geq \epsilon_{2}\right)\leq 2\text{ exp}\left(-2m\cdot \epsilon_{2}^{2}\right).$$

Furthermore, from Lemma 4,

$$Pr\left(\left|\hat{R}(s,t)-\tilde{R}(s,t)\right|\geq \epsilon_{1}\right)=Pr\left(\left|\frac{1}{m}\overset{m}{\underset{i=1}{\sum }}\text{PHS}\left(s,t|P_{i}\right)-\frac{1}{m}\overset{m}{\underset{i=1}{\sum }}\tilde{\text{PHS}}\left(s,t|P_{i}\right)\right|\geq \epsilon_{1}\right)\;\leq Pr\left(\frac{1}{m}\overset{m}{\underset{i=1}{\sum }}\left|\text{PHS}\left(s,t|P_{i}\right)-\tilde{\text{PHS}}\left(s,t|P_{i}\right)\right|\geq \epsilon_{1}\right)$$

$$Pr\left(\left|\hat{R}(s,t)-\tilde{R}(s,t)\right|\leq \epsilon_{1}\right)\geq Pr\left(\frac{1}{m}\overset{m}{\underset{i=1}{\sum }}\left|\text{PHS}\left(s,t|P_{i}\right)-\tilde{\text{PHS}}\left(s,t|P_{i}\right)\right|\leq \epsilon_{1}\right)\;\geq Pr\left(\overset{m}{\underset{i=1}{\cap }}\left|\text{PHS}\left(s,t|P_{i}\right)-\tilde{\text{PHS}}\left(s,t|P_{i}\right)\right|\leq \epsilon_{1}\right)$$

$$Pr\left(\left|\hat{R}(s,t)-\tilde{R}(s,t)\right|\geq \epsilon_{1}\right)\leq Pr\left(\overset{m}{\underset{i=1}{\cup }}\left|\text{PHS}\left(s,t|P_{i}\right)-\tilde{\text{PHS}}\left(s,t|P_{i}\right)\right|\geq \epsilon_{1}\right)\;\leq \sum_{i=1}^{m}Pr\left(\left|\text{PHS}\left(s,t|P_{i}\right)-\tilde{\text{PHS}}\left(s,t|P_{i}\right)\right|\geq \epsilon_{1}\right)\;\leq \sum_{i=1}^{m}4k\left(s,t|P_{i}\right)\text{ exp }\left(-\frac{n\left(s,t|P_{i}\right)}{k\left(s,t|P_{i}\right)}\cdot \frac{\epsilon_{1}^{2}}{c\left(\epsilon_{1}\right)}\right).$$

Hence, for all (*s*, *t*) ∈ *S* × *T*,

$$Pr(\left|\hat{R}(s,t)-R(s,t)\right|\geq \epsilon )\leq 2\text{ exp }\left(-2m\epsilon_{2}^{2}\right)+\overset{m}{\underset{i=1}{\sum }}4k\left(s,t|P_{i}\right)\text{ exp }\left(-\frac{n\left(s,t|P_{i}\right)}{k\left(s,t|P_{i}\right)}\cdot \frac{\epsilon_{1}^{2}}{c\left(\epsilon_{1}\right)}\right)$$

$$Pr\left(\underset{(s,t)\in S\times T}{\cup }\left|\hat{R}(s,t)-R(s,t)\right|\geq \epsilon \right)\leq \underset{(s,t)\in S\times T}{\sum }Pr(\left|\hat{R}(s,t)-R(s,t)\right|\geq \epsilon )\;\leq 2\left|S\right|\left|T\right|\text{exp}\left(-2m\cdot \epsilon_{2}^{2}\right)\;+\underset{(s,t)\in S\times T}{\sum }\overset{m}{\underset{i=1}{\sum }}4k\left(s,t|P_{i}\right)\text{exp}\left(-\frac{n\left(s,t|P_{i}\right)}{k\left(s,t|P_{i}\right)}\cdot \frac{\epsilon_{1}^{2}}{c\left(\epsilon_{1}\right)}\right).$$

Fix *r*_1_, *r*_2_ > 0 such that *r*_1_ + *r*_2_ = *r*. We now see that for

$$n\left(s,t|P_{i}\right)\;=\frac{c\left(\epsilon_{1}\right)\cdot k\left(s,t|P_{i}\right)}{\epsilon_{1}^{2}}\text{ln}\left(\frac{1}{r_{1}}\underset{(s,t)\in S\times T}{\sum }\overset{m}{\underset{i=1}{\sum }}4k\left(s,t|P_{i}\right)\right)\;\leq \frac{c\left(\epsilon_{1}\right)\cdot k\left(s,t|P_{i}\right)}{\epsilon_{1}^{2}}\text{ln}\left(\frac{4m\left|S\right|\left|T\right|k_{\text{max}}}{r_{1}}\right)$$

and

$$m=\frac{1}{2\epsilon_{2}^{2}}\text{ln}\left(\frac{2\left|S\right|\left|T\right|}{r_{2}}\right),$$

we have

$$Pr\left(\underset{(s,t)\in S\times T}{\cup }\left|R(s,t)-\tilde{R}(s,t)\right|\geq \epsilon \right)\leq r.$$

We now notice that the total number of walks taken to run the algorithm (ignoring dead ends) is at most $m\cdot \text{max}\left\{n\left(s,t|P_{i}\right)\right\}=mn$, where $n=\frac{c\left(\epsilon_{1}\right)\cdot k_{\text{max}}}{\epsilon_{1}^{2}}\text{ln}\left(\frac{4m\left|S\right|\left|T\right|k_{\text{max}}}{r_{1}}\right)$. We optimize to minimize *nm* by setting $\epsilon_{1}=\frac{\epsilon }{2}$ and $r_{1}=r\cdot \frac{2m_{0}k_{\text{max}}}{2m_{0}k_{\text{max}}+1}$, where $m_{0}=\frac{1}{2\epsilon_{2}^{2}}\text{ln}\left(\frac{2\left|S\right|\left|T\right|}{r}\right)$ is some approximation for *m*. (We do not claim that these choices are optimal.) □

## Appendix B

### Appendix B.1. Analysis of Just-in-Time (JIT) Dead-End Removal

In our given Pruned HeteSim algorithm, whenever a dead-end node is found, it is removed from the graph for all future walks. We model this as follows. Assume there are m∈ℕ dead-end nodes. Let $w\in {\mathbb{R}}_{\geq 0}$ be the maximum probability of reaching any single dead end. Thus, the probability of reaching a dead end is at most *mw*. Let $\alpha \in {\mathbb{R}}_{\geq 0}$ be the probability of any given walk not ending in a dead end and let *β* = *mw* + *α*.

We now analyse the number of non-dead-end walks we expect to take by the time we hit some fixed number of dead ends and the number of dead ends we expect to take by the time we hit some fixed number of non-dead ends.

In the JIT algorithm, whenever we hit a dead end, the probability of hitting a dead end in the future is affect as follows. Let *X*_1_, ··· ∈ {0, 1}, where *X*_*i*_ = 1 if the *i*th walk is not a dead end and *X*_*i*_ = 0 otherwise. For all *i*,

$$Pr\left(X_{i}=1\right)=\frac{\alpha }{\beta -wY_{i}^{\prime }},$$

where *Y*_*i*_ is the number of *X*_*j*_ = 0 for *j* < *i*. (Thus, treating *w* as the weight of each dead end and *α* as the weight on non-dead ends, each time we hit a dead end, the weight of the dead end hit is lost as we can no longer get to that dead end. This means that overtime the probability of hitting a dead end decreases.)

Let *S*_*i*_ be the number of *X*_*j*_ = 1 before the *i*-th *X*_*j*_ = 0. Let *T*_*i*_ be the number of *X*_*j*_ = 0 before the *i*-th *X*_*j*_ = 1.

**Theorem A1**. *For all* i∈ℕ,

$$S_{i}=\overset{i-1}{\underset{j=0}{\sum }}{\text{Z}}_{j}-i,$$

*where Z*_*j*_ *is geometrically distributed with parameter $\frac{(m-j)w}{\beta -wj}$*.

**Proof**. Notice that after the *k*th *X*_*j*_ = 0, the probability of *X*_*j*_ = 1 is $\frac{(m-k)w}{\beta -wk}$ (the probability of *X*_*j*_ = 0 after that point). Between the *k*-th *X*_*j*_ = 0 and (*k* + 1)-th *X*_*j*_ = 0, this probability is fixed. Thus, the number of *X*_*j*_ = 1 between the *k*-th *X*_*j*_ = 0 and (*k* + 1)-th *X*_*j*_ = 0 is geometrically distributed with parameter $\frac{(m-k)w}{\beta -wk}$. (We subtract *i* to not count the *X*_*j*_ = 0). □

**Theorem A2**.

$$E\left(S_{i}\right)=\frac{\alpha }{w}\overset{i-1}{\underset{j=0}{\sum }}\frac{1}{m-j}$$

*and*

$$Var\left(S_{i}\right)=\frac{\alpha }{w^{2}}\overset{i-1}{\underset{j=0}{\sum }}\frac{\alpha +w(m-j)}{{\left(m-j\right)}^{2}}.$$

**Proof**. Follows from linearity of expectation and standard results about the geometric distribution. □

**Remark A1**. *We can obtain a bound of the deviation from the mean using Chebyshev’s inequality*.

**Lemma A6**. *For i* ≥ 2 *and k*_*i*−1_ ≤ *k*_*i*_ ≤ *m*,

$$Pr\left(T_{i}=k_{i}|T_{i-1}=k_{i-1}\right)=\frac{\alpha }{\beta -wk_{i}}\cdot \overset{k_{i}-1}{\underset{t=k_{i-1}}{\prod }}\frac{(m-t)w}{\beta -tw}$$

*and*

$$Pr\left(T_{1}=k_{1}\right)=\frac{\alpha }{\beta -wk_{1}}\cdot \overset{k_{1}-1}{\underset{t=0}{\prod }}\frac{(m-t)w}{\beta -tw}.$$

**Proof**. The distribution is similar to a geometric distribution with the change that for each failure the probability of success changes. □

We now see that

$$Pr\left(T_{n}=k_{n},\cdots ,T_{1}=k_{1}\right)=Pr\left(T_{1}=k_{1}\right)\overset{n}{\underset{i=2}{\prod }}Pr\left(T_{i}=k_{i}|T_{i-1}=k_{i-1}\right)\;=\alpha^{n}\left(\overset{n}{\underset{i=1}{\prod }}\frac{1}{\beta -wk_{i}}\right)\cdot \left(\overset{k_{n}-1}{\underset{t=0}{\prod }}\frac{(m-t)w}{\beta -tw}\right)$$

$$Pr\left(T_{n}=k_{n}\right)={\left(\frac{\alpha }{\beta }\right)}^{n}\left(\overset{k_{n}-1}{\underset{t=0}{\prod }}\frac{(m-t)w}{\beta -tw}\right)\overset{k_{n}}{\underset{k_{n-1}=0}{\sum }}\cdots \overset{k_{2}}{\underset{k_{1}=0}{\sum }}\left(\overset{n}{\underset{i=1}{\prod }}\frac{1}{1-\frac{w}{\beta }k_{i}}\right).$$

**Lemma A7**. *For all n* ≥ 1,

$$\overset{k_{n}}{\underset{k_{n-1}=0}{\sum }}\cdots \overset{k_{2}}{\underset{k_{1}=0}{\sum }}\left(\overset{n}{\underset{i=1}{\prod }}\frac{1}{1-\frac{w}{\beta }k_{i}}\right)\leq \frac{{\left(-1\right)}^{n-1}}{(n-1)!}{\left(\frac{\beta }{w}\right)}^{n-1}\frac{\text{ln}{\left(1-\frac{w}{\beta }\left(k_{n}+n-1\right)\right)}^{n-1}}{1-\frac{w}{\beta }k_{n}}.$$

**Proof**. We note that for increasing *f*,

$$\int_{a-1}^{b}f(x)\;dx\leq \overset{b}{\underset{k=a}{\sum }}f(k)\leq \int_{a}^{b+1}f(x)dx.$$

We prove this result inductively. Assume the result holds for *n* = *m*. For the upper bound, we now see that

$$\overset{k_{m+1}}{\underset{k_{n-1}=0}{\sum }}\cdots \overset{k_{2}}{\underset{k_{1}=0}{\sum }}\left(\overset{m+1}{\underset{i=1}{\prod }}\frac{1}{1-\frac{w}{\beta }k_{i}}\right)\;=\frac{1}{1-\frac{w}{\beta }k_{m+1}}\overset{k_{m+1}}{\underset{k_{m}=0}{\sum }}\left(\overset{k_{m}}{\underset{k_{m-1}=0}{\sum }}\cdots \overset{k_{2}}{\underset{k_{1}=0}{\sum }}\left(\overset{m}{\underset{i=1}{\prod }}\frac{1}{1-\frac{w}{\beta }k_{i}}\right)\right)\;\leq \frac{1}{1-\frac{w}{\beta }k_{m+1}}\cdot \frac{1}{(m-1)!}{\left(\frac{\beta }{w}\right)}^{m-1}\overset{k_{m+1}}{\underset{k_{m}=0}{\sum }}\frac{{\left(-1\right)}^{m-1}\text{ln}{\left(1-\frac{w}{\beta }\left(k_{m}+m-1\right)\right)}^{m-1}}{1-\frac{w}{\beta }k_{m}}.$$

We note that

$$\frac{{\left[-\text{ln}\left(1-\frac{w}{\beta }\left(k_{m}+m-1\right)\right)\right]}^{m-1}}{1-\frac{w}{\beta }k_{m}}$$

is increasing as a function in *k*_*m*_ and positive for *k*_*m*_ ≥ 0 for all m∈ℕ. Hence,

$$\sum_{k_{n-1}=0}^{k_{m+1}}\cdots \sum_{k_{1}=0}^{k_{2}}\left(\prod_{i=1}^{m+1}\frac{1}{1-\frac{w}{\beta }k_{i}}\right)\;\leq \frac{1}{1-\frac{w}{\beta }k_{m+1}}\cdot \frac{{\left(-1\right)}^{m-1}}{(m-1)!}{\left(\frac{\beta }{w}\right)}^{m-1}\int_{0}^{k_{m+1}+1}\frac{\text{ln}{\left(1-\frac{w}{\beta }(x+m-1)\right)}^{m-1}}{1-\frac{w}{\beta }x}dx\;\leq \frac{1}{1-\frac{w}{\beta }k_{m+1}}\cdot \frac{{\left(-1\right)}^{m-1}}{(m-1)!}{\left(\frac{\beta }{w}\right)}^{m-1}\int_{0}^{k_{m+1}+1}\frac{\text{ln}{\left(1-\frac{w}{\beta }(x+m-1)\right)}^{m-1}}{1-\frac{w}{\beta }(x+m-1)}dx\;\leq \frac{{\left(-1\right)}^{m}}{m!}{\left(\frac{\beta }{w}\right)}^{m}\frac{\left[\text{ln}{\left(1-\frac{w}{\beta }\left(k_{m+1}+m\right)\right)}^{m}-\text{ln}{\left(1-\frac{w}{\beta }(m-1)\right)}^{m}\right]}{1-\frac{w}{\beta }k_{m+1}}\;\leq \frac{{\left(-1\right)}^{m}}{m!}{\left(\frac{\beta }{w}\right)}^{m}\frac{\text{ln}{\left(1-\frac{w}{\beta }\left(k_{m+1}+m\right)\right)}^{m}}{1-\frac{w}{\beta }k_{m+1}}.$$

□

**Theorem A3**.

$$Pr\left(T_{n}=k_{n}\right)\leq {\left(\frac{\alpha }{w}\right)}^{n}\left(\overset{k_{n}-1}{\underset{t=0}{\prod }}\frac{(m-t)w}{\beta -tw}\right)\frac{{\left(-1\right)}^{n-1}}{(n-1)!}\cdot \frac{\text{ln}{\left(1-\frac{w}{\beta }\left(k_{n}+n-1\right)\right)}^{n-1}}{\frac{\beta }{w}-k_{n}}.$$

**Proof**. Follows from Lemma A7. □
